# Cognitive mechanisms of learning in sequential decision-making under uncertainty: an experimental and theoretical approach

**DOI:** 10.3389/fnbeh.2024.1399394

**Published:** 2024-08-12

**Authors:** Gloria Cecchini, Michael DePass, Emre Baspinar, Marta Andujar, Surabhi Ramawat, Pierpaolo Pani, Stefano Ferraina, Alain Destexhe, Rubén Moreno-Bote, Ignasi Cos

**Affiliations:** ^1^Facultat de Matemàtiques i Informàtica, Universitat de Barcelona, Barcelona, Spain; ^2^Center for Brain and Cognition, DTIC, Universitat Pompeu Fabra, Barcelona, Spain; ^3^CNRS, Institute of Neuroscience (NeuroPSI), Paris-Saclay University, Saclay, France; ^4^Department of Physiology and Pharmacology, Sapienza University of Rome, Rome, Italy; ^5^Serra-Hunter Fellow Programme, Barcelona, Spain

**Keywords:** decision-making, learning, cognition, computational modeling, consequence, uncertanty, neural dynamics, behavior

## Abstract

Learning to make adaptive decisions involves making choices, assessing their consequence, and leveraging this assessment to attain higher rewarding states. Despite vast literature on value-based decision-making, relatively little is known about the cognitive processes underlying decisions in highly uncertain contexts. Real world decisions are rarely accompanied by immediate feedback, explicit rewards, or complete knowledge of the environment. Being able to make informed decisions in such contexts requires significant knowledge about the environment, which can only be gained via exploration. Here we aim at understanding and formalizing the brain mechanisms underlying these processes. To this end, we first designed and performed an experimental task. Human participants had to learn to maximize reward while making sequences of decisions with only basic knowledge of the environment, and in the absence of explicit performance cues. Participants had to rely on their own internal assessment of performance to reveal a covert relationship between their choices and their subsequent consequences to find a strategy leading to the highest cumulative reward. Our results show that the participants’ reaction times were longer whenever the decision involved a future consequence, suggesting greater introspection whenever a delayed value had to be considered. The learning time varied significantly across participants. Second, we formalized the neurocognitive processes underlying decision-making within this task, combining mean-field representations of competing neural populations with a reinforcement learning mechanism. This model provided a plausible characterization of the brain dynamics underlying these processes, and reproduced each aspect of the participants’ behavior, from their reaction times and choices to their learning rates. In summary, both the experimental results and the model provide a principled explanation to how delayed value may be computed and incorporated into the neural dynamics of decision-making, and to how learning occurs in these uncertain scenarios.

## 1 Introduction

The brain mechanisms involved in decision-making have been extensively studied in the last decades [reviewed in (Gold and Shadlen, 2007; Wang, 2008)]. Many studies focused on characterizing the neural dynamics of reward processing (Padoa-Schioppa, 2011; Wallis and Kennerley, 2011; Gluth et al., 2014), visual discrimination (Shadlen and Newsome, 1996; Shadlen and Newsome, 2001; Roitman and Shadlen, 2002), and other aspects of option assessment during value-based decision-making (Pastor-Bernier and Cisek, 2011; Wallis, 2011; Cai and Padoa-Schioppa, 2019; Carroll et al., 2019). Other tasks were developed to study decisions in the context of short-term memory (Siegel et al., 2009), and cost-risk trade-off (Kahneman and Tversky, 1979; Birnbaum, 2008; Eichberger and Pasichnichenko, 2021). In most of these contexts, choice outcomes are immediately experienced. This feature makes calculating costs and benefits straightforward, as all the necessary information is directly and immediately available to the decision maker for calculation (Kurniawan et al., 2013; Skvortsova et al., 2014; Apps et al., 2015; Thura and Cisek, 2016). However, a complete account of value-based choice behavior requires understanding the brain mechanisms underlying the detection and computation of non-immediate consequences of choices, and of the use of this information to guide subsequent decision strategies. Despite the rich literature in cognitive decision-making and the fact that long-term consequence is a critical concern in our daily decision-making processes, the dynamics of its operation are not fully understood, and have not been incorporated into state-of-the-art models of decision-making (Brunel and Wang, 2001; Wong and Wang, 2006; Wong et al., 2007). Most previous models work only for independent trials by considering value and/or accumulation of evidence about choice alternatives (Drugowitsch et al., 2012). They often do not, however, take into consideration the memory of recent past or the long-term effects of decisions in the context of brain dynamics. By contrast, studies on hierarchical decision-making show that when choices are repeatedly made along nodes of the same decision-tree, they tend to integrate elements of subsequent nodes (Hyafil and Moreno-Bote, 2017). In other words, the assessment of options during decisions incorporates elements of subsequent branching points. However, for these decisions to be informed, exploration and ultimately knowledge about future nodes is required.

Here we are interested in formalizing the brain mechanisms underlying how this exploration leads to information gain when the strategy is non-obvious. In other words, which are the brain operations involved in considering the consequence of choices during sequences of decisions. In this scenario, the case when the immediate most rewarding choice leads to lower long-term reward is of particular interest, as participants must anticipate that the cost of choosing lower value options results in increased delayed reward and higher cumulative reward overall. Moreover, if this relationship is covert, what are the cognitive mechanisms that enable us to learn the optimal strategy? Furthermore, how does the learning occur in the absence of explicit performance feedback?

To answer these questions, we developed the *consequential task*. Consecutive perceptual decision-making trials were organized into groups of dependent trials, where the choice made in one trial had a consequence on the next by determining the available choice options. How does the complexity of a perceptual decision-making task augment when combined with consequence assessment? First, consequence-based decisions (i.e., decisions in which optimal performance can only be achieved after acquiring knowledge of future nodes) require an increased temporal span of consideration, and, consequently, involve a more uncertain and broader set of factors to examine. This typically results in more computationally demanding option evaluation (Trommershäuser et al., 2008; Nagengast et al., 2011; O’Brien and Ahmed, 2015; Kirchler et al., 2017), longer deliberation, and often poorer decision accuracy (Schuck-Paim and Kacelnik, 2007; Drugowitsch et al., 2016). Second, making decisions based on gauging choice consequence involves a range of cognitive processes broader than those involved in immediate sensory-motor decisions (Cisek et al., 2009; Donner et al., 2009), with particular emphasis on value integration (Cisek and Kalaska, 2005; Park et al., 2011), metacognitive processing (Klaes et al., 2011; Goodwin et al., 2012) and long-term working memory (Cavanagh et al., 2018; Barbosa et al., 2020). Though long-term consequence assessment may be viewed as a time extended version of immediate action outcome evaluation, significant doubts remain regarding the core cognitive and neural processes underlying this ability (Balasubramani and Hayden, 2018).

To investigate the cognitive processes underlying consequence-based decision-making, we carried out a combined experimental and theoretical study. In the first part of this work, we designed a decision-making task, the *consequential task*, to characterize consequence-based option assessment. In brief, in the consequential task, consequence takes the form of increases/decreases in future reward value options as a function of participants’ choices. The nature of this inter-trial dependence was not disclosed in the instructions given to the participants, and no explicit performance feedback was provided. The absence of explicit learning cues was intended to force the participants to rely on their own subjective performance feedback to infer the delayed consequence of their decisions.

In the second part of our study, we provided a theoretical framework of the cognitive and neural processes required for consequence-based decision-making, including the patterns of inhibition and of far-sighted consequence assessment required to acquire the most reward across trials. The model was organized in three layers. The bottom layer, in line with the Amari, Wilson-Cowan and Wong-Wang models (Wilson and Cowan, 1972; Soltani et al., 2006; Wong and Wang, 2006; Webb et al., 2011; Marcos et al., 2013; Hertäg et al., 2014), described the neural dynamics of binary decision-making by means of two populations of neurons. The middle and top layers modeled an oversight mechanism for the assessment of consequence across groups of trials and the learning mechanism as a function of reward value across trials. This model reproduced the full range of behavioral observations across the different participants accurately while predicting a plausible neural implementation of the processes underlying the learning of consequence-based decision-making. In particular, our model described how the metacognitive assessment of consequence extends from short to long-term value prediction through an oversight mechanism that monitors predicted performance.

## 2 Results

### 2.1 Task design

**Figure 1:**
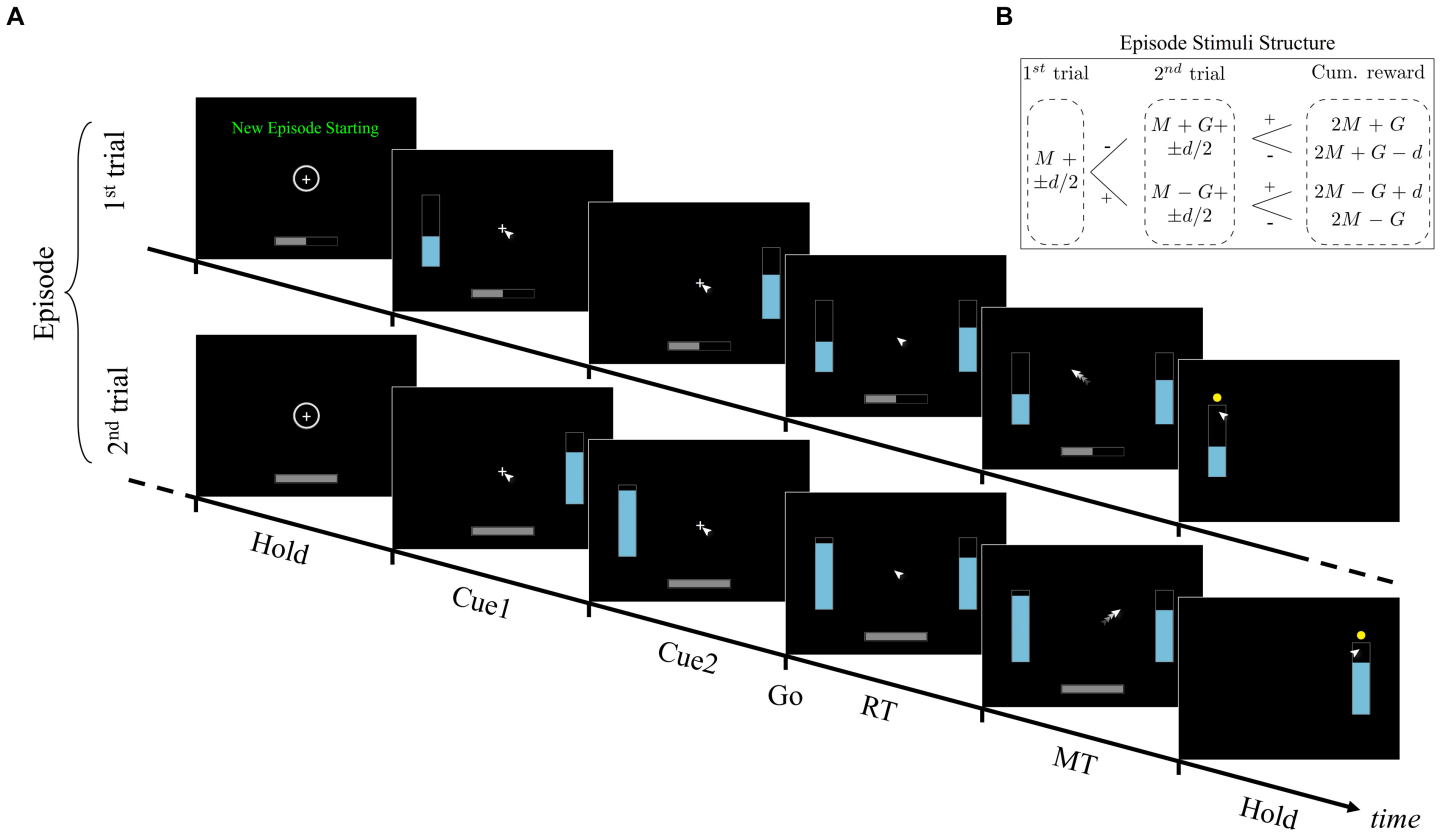
Time-course of a typical horizon 1 episode of the consequential decision-making task. **(A)** The episode consists of two dependent trials. The first starts with the message “New Episode Starting” in the center-top of the screen, a circle surrounding a cross in the center (central target), and a half full progress bar at the bottom of the screen. The progress bar indicates the current trial within the episode (for horizon 1, 50% during the first trial, 100% during the second trial). After holding for 500 ms, the left or right (chosen at random) stimulus is shown, followed by its complementary stimulus 500 ms later. Both stimuli are shown simultaneously 500 ms later which serves as the GO signal. At GO, the participant has to slide the mouse from the central target to the bar of their choosing. Once the selected target is reached, a yellow dot appears over that target. The second trial follows the same pattern as the first. See Methods for more details. **(B)** Construction scheme for the size of the stimuli in each episode. The first trial within the episode consists of 2 stimuli of size M + d/2 and M−d/2. The second trial within the episode depends on the selection made in the previous trial. If the first selected stimulus is M−d/2 (following symbol “-” in the figure), then the second trial consists of stimuli with size M + G + d/2 and M + G−d/2, otherwise M-G + d/2 and M-G-d/2 (following symbol “+” in the figure). The cumulative reward value of the episode can therefore assume 4 distinct values (ordered from best to worst): 2 M + G, 2 M + G-d, 2 M-G + d, and 2 M-G. See Methods for more details on the values of M, G, d.

In this section we describe the consequential task and, more specifically, how it is designed to tap into the cognitive mechanisms involved in learning delayed consequences in the absence of explicit performance feedback. In this task, 28 healthy participants were instructed to choose one of two stimuli presented left and right on a screen. The stimuli represented partially filled containers of water and reward value was directly proportional to the amount of water in each container. The participants reported their choices by moving the computer mouse’s cursor from the central cue to the chosen stimulus (see Figure 1 and Materials and Methods for a thorough description). Participants were only paid a show-up fee and were, thus, not monetarily incentivized to perform well.

Since consequence depends on a predictive assessment of future contexts, the task was organized into two block types. In the first, trials required one-shot decisions, purely independent of one another. Similar to most decision-making tasks, the reward value in this block type could be maximized by picking the stimulus associated with the most reward value in each trial, i.e., choosing the larger of the two blue bars. However, in the second block type, trials were grouped into pairs or triads of dependent trials. We called each group of consecutive trials an episode to signify the boundary of dependence between them, and defined the notion of horizon (*n_H_*) as a metric for the depth of consequence to be expected for that episode. In other words, *n_H_* equaled the number of dependent trials following the first trial of the episode. For example, for *n_H_* = 1 an episode consisted of 2 trials, with the second depending on the first. The nature of the dependence between trials of an episode was such that the mean reward values of the stimuli in the second/third trial were systematically increased or decreased based on the participant’s choice in the preceding trial. Choosing the larger stimulus value led to a reduction of stimuli values in the subsequent trial whereas choosing the smaller stimulus in the first trial led to an increase (Figure 1B). The increment/reduction amount (*G*) was a constant and chosen such that selecting the larger stimulus in the first trial could never compensate for the loss in future reward value. In other words, acquiring the maximum cumulative reward value in each episode required choosing “big” in single trial episodes (horizon *n_H_* = 0), and choosing “small” in all trials of *n_H_* = 1 and *n_H_* = 2 episodes except the last, in which “big” should be chosen.

The consequential task design enables investigation into the role of perceived consequence during sequential decision-making. Consequence, in this context, refers to the influence of a choice on the stimuli values in the trial next. The post-decision stimuli heights function as a form of feedback which participants must learn to interpret in order to become aware of and evaluate the consequences associated with particular choices. Performance feedback, however, is absent from the task in that participants are never presented with cues indicating whether they are behaving optimally. This absence required participants to evaluate their own performance based on their experience during task execution. Importantly, participants were not informed of the nature of the inter-trial dependence and had to discover it on their own via exploration. Explicit performance feedback might have had the undesirable effect of participants focusing on finding the specific sequence of choices yielding optimal performance feedback, without having to learn the dependence between their decisions and the subsequent trials. In other words, an explicit measure of performance might have reduced the task to an explicit trial-and-error test in which participants would experiment with different sequences of choices (“big-small,” “small-big,” etc.) until finding the sequence leading to maximum performance, rather than learning to evaluate each option’s consequence in terms of their prediction of future reward. In contrast, the absence of performance feedback obligated participants to create an internal sense of assessment, which could only rely on two mechanisms: the sensory perception of the systematic stimuli changes in the subsequent trial after each choice, and the exploration of option choices at each trial during the earlier part of each block. The resulting task essentially becomes a measure of learning about delayed consequences associated with each option in the absence of explicit performance feedback.

In summary, for the participants to be able to perform the task, they were informed of the episode-based organization of trials at each block, i.e., the horizon. The instruction to the participant was to find the strategy leading to the most cumulative reward value for each episode and to actively explore their choices. Learning the optimal policy was challenging due to several factors. First, perceptual discrimination was difficult in some trials since the height difference between stimuli could be as low as 1% the height of the container. Second, although participants were informed that their choices may affect future trials within the episode, the nature of this dependency was not specified. This means that from the perspective of the participants, the value of the stimuli offers might at first appear random. Third, explicit performance feedback was omitted from the task after each episode, requiring participants to discover the nature of the inter-trial dependencies via exploration. Further details are shown in the Methods section, and in Figure 1.

### 2.2 Behavioral results

Several metrics were extracted from the participants’ behavioral data: performance (PF), reported choices (CH), reaction time (RT), and visual discrimination (VD) sensitivity. PF was extracted from each episode and assumed values between 0 (worst) and 1 (best). PF was calculated as the percentage of the maximum possible reward value acquired in each episode and is normalized such that PF = 0 in episodes wherein the participant acquired the minimum possible reward value. CH was the choice made by the participant in each trial and could take one of two values: small (i.e., smaller stimulus), or large (i.e., larger stimulus). RT was calculated as the time difference between the simultaneous presentation of both stimuli (the GO signal), and the onset of the movement. VD is a measure of each participants’ ability to visually discriminate between stimuli, i.e., identifying which stimulus is bigger/smaller (see Methods for further details). As shown below, when the difference between stimuli (ΔS) is the smallest, participants were not able to accurately distinguish between stimuli. The ΔS varies between 1 and 20% of the size of the container. Note that for horizon *n_H_ = 0,* a trial with ΔS = 0.01 is perceptually difficult, but if chosen wrong, the difference in the final reward would be small (1%). However, for horizon *n_H_ = 1 or 2*, choosing the wrong stimulus due to perceptual discrimination has a large impact on the final performance, since it leads to a decrease of the available stimuli in the next trials.

The absence of explicit performance-related feedback at the end of each episode made the task more difficult, and, consequently, not all participants were able to find the optimal strategy. For horizon *n_H_ = 0,* 26 of the 28 participants learned and applied the optimal strategy, i.e., repeatedly selecting the larger stimulus. In contrast, only 22 participants learned the optimal strategy during horizon *n_H_ = 1, 2* blocks, i.e., selecting the larger stimulus in the last trial only.

We analyzed the exploratory strategies the participants employed. In particular, we tested whether participants only considered the size of the stimuli (small/big), or if they also tested other hypotheses involving the order of presentation of the stimuli (first/s) or the location (left/right) of the stimuli. The result of this analysis can be found in the Supplementary Figure S1. In brief, participants’ choices overwhelmingly depended on stimuli size and there was little evidence other factors such as order of presentation or location were seriously considered in the decision-making process. Most participants who did not learn the optimal strategy for *n_H_ = 1,2* repeatedly chose the larger stimulus for all trials.

In Materials and Methods (subsection Consequential Decision-Making task), we described how the task was structured, and we mentioned that we randomized the order in which participants performed the horizons. This means that, for example, some participants performed *n_H_* = 2 before *n_H_* = 0. We wondered if the order of execution of the horizons had an influence on learning. To address this, we performed an analysis comparing learning times for different orders of horizon presentation. The results of this investigation can be found in the Supplementary Figures S2, S3. In brief, we discovered that once the optimal strategy was understood in *n_H_* = 1 or 2, participants generalized the rule and, by abstraction, applied it to the horizon performed afterwards. For this reason, we defined a single learning time per session. We defined *learning time (t_L_)* as the number of episodes that occurred before the optimal strategy was assimilated. We considered the optimal strategy to be assimilated if the participant employed it in at least 9 out of the following 10 episodes, and 75% of the remaining episodes until the end of the block. To account for perceptual discrimination errors (during low VD), we excluded the most difficult episodes in terms of ΔS to calculate the learning time.

**Figure 2:**
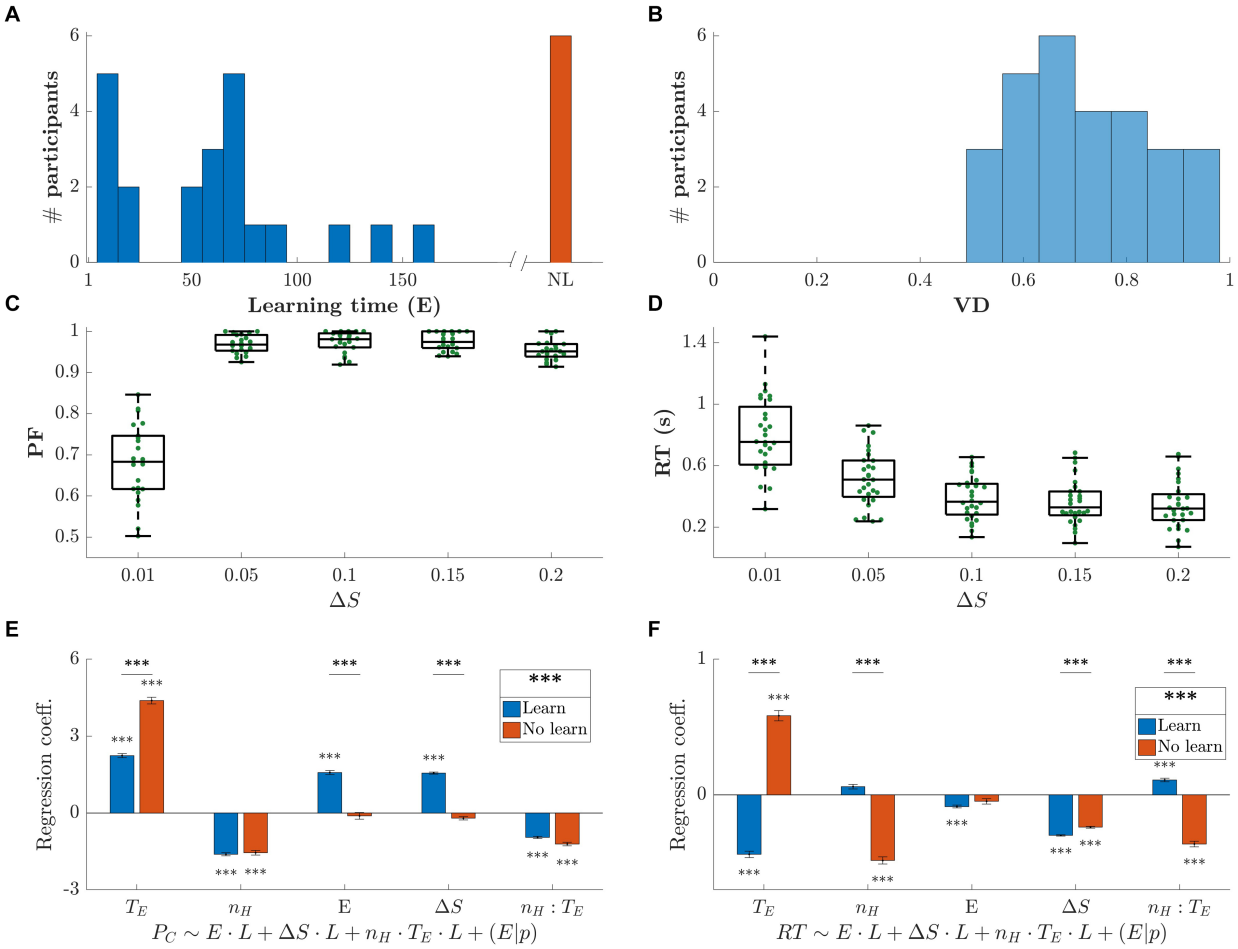
Summary behavioral results across participants. **(A)** Histogram of learning times. Learning time is defined as the number of episodes **(E)** throughout the whole session before the optimal strategy was applied repeatedly (see Methods). We identified four groups of participants: fast, medium and slow learners, and participants who did not discover the optimal strategy (NL – No Learning). **(B)** Histogram of visual discrimination (VD) calculated by computing the percentage of correct selections of the last 80 episodes, in the horizon 0 block, for only the most difficult trials (Δ*S* = 0.01). **(C)** Performance as a function of ΔS, for the trials after the optimal strategy was applied. **(D)** Reaction Time (RT) versus Δ*S*. The more similar the stimuli, the longer participants needed to make a decision. **(E,F)** Regression coefficients for the generalized linear mixed-effects models $P_{oc}\sim L\cdot E+L\cdot \Delta S+L\cdot n_{H}\cdot T_{E}+(E|p)$ and $RT\sim L\cdot E+L\cdot \Delta S+L\cdot n_{H}\cdot T_{E}+(E|p)$ , where $P_{oc}$ is the probability of making the optimal choice, RT is the reaction time, E is the episode number, $n_{H}$ is the horizon number, $T_{E}$ is the trial within episode, L identifies the group of participants that learned the optimal strategy, $n_{H}:T_{E}$ is the interaction term, and p is the participant. We used maximum likelihood to estimate the model parameters. Participants were divided into two groups: those who learned the optimal strategy (blue) and those who did not (red), see Panel (a). The statistical difference between learning groups in reported next to the legend.

Figure 2 shows the summary results for all 28 participants. In Panel (a), we show the histogram of their learning times in terms of episodes (*E*). The last histogram bar in Figure 2A (shown as NL – No Learning) represents the 6 participants who never learned the optimal strategy. We divided participants into 4 groups as a function of their learning speed: slow, medium, fast, and those who never learned the optimal strategy.

Figure 2B shows the VD, for all difficult trials (smallest ΔS) and participants, where VD was calculated as the percentage of correct choices over the last 80 episodes in the horizon *n_H_ = 0* block. On average, stimuli were discriminated correctly in 71% of the most difficult trials. This indicates that most participants continued making errors after learning the optimal strategy due to low VD. This is reported in Figure 2C which shows the grand average and standard error of the PF across subjects as a function of the difficulty level for all episodes following each participant’s learning time (*p* = 10^−12^, F-stat = 59). Note that, in Figure 2D, RT increased as a function of VD (*p* = 10^−25^, F-stat = 160).

The dependence of PF and RT on VD together with the other variables had to be established statistically. To assess the learning process, we quantified the relationship of PF and RT with horizon *n_H_*, trial within episode *T_E_*, and episode *E*. To obtain consistent results, we adjusted these variables as follows. The trial within episode was reversed chronologically, because the optimal choice for the last *T_E_* (large) is the same regardless of the horizon number. Furthermore, regarding the model of PF, we made a per trial adaptation of PF (PF was originally calculated per episode), i.e., the probability of choosing the optimal choice $P_{oc}$ . Finally, to assess differences between learning groups, we introduced the categorical variable L that identified the group of participants that learned the optimal strategy and the ones who did not (as seen in Figure 2A). We then used a generalized linear mixed effects model (Verbeke and Molenberghs, 2009; Gałecki and Burzykowski, 2013) to predict PF and RT. The independent variables for the fixed effects are horizon *n_H_*, trial within episode $T_{E}$ , the passage of time expressed in terms of episodes *E,* and ΔS. We set the random effects for the intercept and the episodes grouped by participant *p*; we write the random effects as (E|p) . The resulting models are: $P_{oc}\sim L\cdot E+L\cdot \Delta S+L\cdot n_{H}\cdot T_{E}+(E|p)$ and $RT\sim L\cdot E+L\cdot \Delta S+L\cdot n_{H}\cdot T_{E}+(E|p).$ The regression coefficients, along with their respective group significance, are shown in Figures 2E,F. The detailed results of the statistical analysis are reported in Section 5.5. In panel (e), $P_{oc}$ increases with $T_{E}$ , suggesting that the first trial(s) within the episode are less likely to be guessed right, i.e., choosing the smaller stimuli. This makes sense, since only the early trials within episode required inhibition. Moreover, looking at the amplitude of the regression coefficients, we can see that this effect is even stronger in the no-learning case. The same argument can be made for the dependence with *n_H_*. A strong difference between learning and no-learning can be appreciated when considering the time dependence: for the learners group $P_{oc}$ increases as time goes by, i.e., *E* increases, while it is not significant for the group that did not learn the optimal strategy. The two learning groups are globally statistically different (*p = 10^−7^*). In panel (f), RT shows converse effect directions between learning and no-learning groups for both dependencies on $T_{E}$ and *n_H_*. The participants who learned the optimal strategy exhibited longer RT for the earlier trials within the episode, consistent with the need to inhibit the selection of the larger stimulus. Also, the larger the horizon, the longer the RT, opposite to the no-learning group. As expected, RT increases with decreasing ΔS for both groups. The two learning groups are globally statistically different (*p = 10^−17^*).

**Figure 3:**
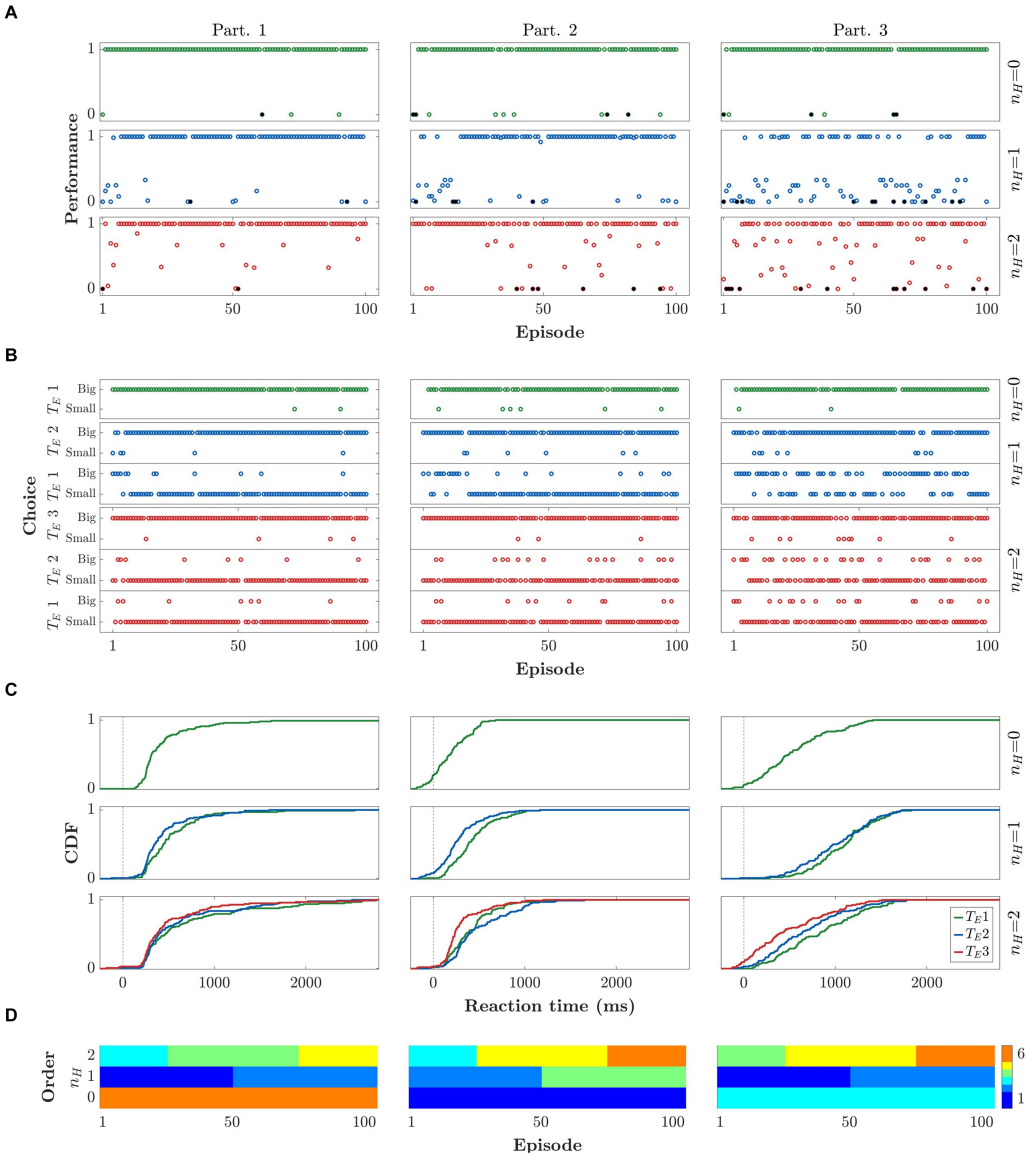
Behavioral results for three representative participants. Rows and columns refer to horizons (*n*_*H*_) and participants, respectively. **(A)** Performance per episode. **(B)** Choice behavior per trial, in terms of selecting the bigger or smaller stimulus. Results are gathered by horizon (*n*_*H*_) and respective trial within episode (*T*_*E*_). **(C)** Cumulative density function (CDF) of reaction times. The color code indicates the trial within episode (green for *T*_*E*_ = 1, blue for *T*_*E*_ = 2, and red for *T*_*E*_ = 3). **(D)** Order of execution of blocks and horizons.

Figure 3 depicts the data from 3 sample participants. In particular we show their PFs, CHs, and RTs metrics, and the order of execution of the different blocks and horizons. Each column corresponds to a participant and each row to a different horizon level. Note that all three participants performed the *n_H_ = 0* task correctly (Figures 3A,B). The first 2 participants also performed *n_H_ = 1* correctly, while participant 3 did not learn the correct strategy until executing *n_H_ = 2.* Note that participants 1 and 2 performed *n_H_ = 1* before *n_H_ = 2* and were able to apply what they learned in *n_H_ = 1* to *n_H_ = 2.* Because of this, a very fast learning process can be seen during the first *n_H_ = 2* block. In Figure 3C, note that some RTs are negative. In these cases, the participant did not wait for the presentation of the GO signal to start the movement.

### 2.3 A Neurally-inspired model of consequential decision-making

In this section we describe our mathematical formalization of consequential decision-making which incorporates a variable foresight mechanism and adapts to the distribution of reward value across trials. The formalization takes the form of a three-layer neural model. In brief, the bottommost layer is a mean-field model for binary decision-making. The mean-field is driven by a strategy learning layer which then dictate the choices to the decision-making process.

We feel this novel approach yields several advantages over more classical models (i.e., reinforcement learning, drift-diffusion, urgency-gating, etc.). In brief, we aim to provide a formalization of the neural processes involved in reward-driven, delayed-value, multi-step decisions in a context in which attaining reward is contingent on learning the covert effect of actions on the environment. In other words, learning must operate in the absence of explicit performance feedback. Another unique aspect of our approach is the incorporation of a foresight mechanism which adapts to the covert relationship between actions and their effect on the environment as well as to the distribution of reward value across the trials of an episode. We expand on the reasoning behind the creation of our novel formalization in the Discussion section.

#### 2.3.1 Layer 1: Neural dynamics

To describe the neural dynamics at each trial, we used a mean-field approximation of a biophysically based binary decision-making model (Wilson and Cowan, 1972; Brunel and Wang, 2001; Wang, 2002; Thura et al., 2022). This approximation is often used to analyze neuronal dynamics in contexts where mean population activity is relevant. It has been shown that even simple mean-field approximations leveraging as little as two internal variables could reproduce most features of the underlying spiking neuron model (Wong and Wang, 2006).

**Figure 4:**
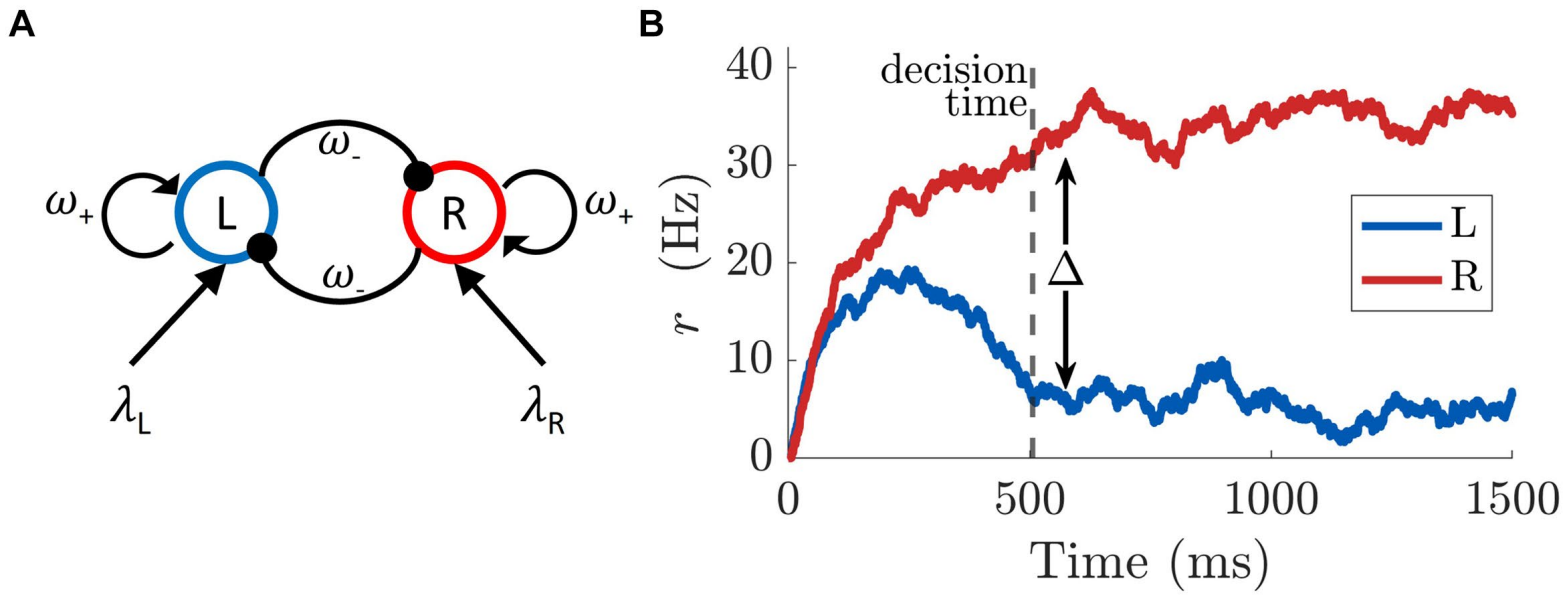
**(A)** Network structure of binary decision model of mean-field dynamics. The L pool is selective for the stimulus L (λ_L_), while the other population is sensitive to the appearance of the stimulus R (λ_R_). The two pools mutually inhibit each other (ω_−_) and have self-excitatory recurrent connections (ω_+_). **(B)** Firing rate of the two populations (L, R) of excitatory neurons according to the dynamics in Eq. 1. A decision is taken at time 506 ms (vertical dashed line) when the difference in activity between L and R pools passes the threshold of Δ =25 Hz. The strengths of the stimuli are set to *λ*_L_ = 0.0203 and *λ*_R_ = 0.0227. The time constant and the noise are set to *τ* = 80 ms and *σ* = 0.003 ms^−1^, respectively.

The core of the model consists of two populations of excitatory neurons: one sensitive to the stimulus on the left-hand side of the screen (L), and the other to the stimulus on the right (R). The intensity of the evidence is the size of each stimulus, which is directly proportional to the amount of reward displayed. In the model this is captured by the parameters λ_L_, λ_R,_ respectively. Though distinguishing between the bigger and smaller stimulus values is critical in our task, in the model it is convenient to characterize stimuli based on their position, i.e., left/right. The reason being that the information regarding target size is already conveyed by the respective stimuli values, i.e., the parameters λ_L_, λ_R_. Moreover, this allows us to introduce an extra degree of freedom in the model without increasing the number of variables. The equations


(1) $$\{\begin{array}{c} \tau \frac{dr_{L}\left(t\right)}{dt}=-r_{L}\left(t\right)+f\left(\lambda_{L}+\omega_{+}r_{L}\left(t\right)-\omega_{-}r_{R}\left(t\right)\right)+\sigma \xi_{L}\left(t\right)\\ \tau \frac{dr_{R}\left(t\right)}{dt}=-r_{R}\left(t\right)+f\left(\lambda_{R}+\omega_{+}r_{R}\left(t\right)-\omega_{-}r_{L}\left(t\right)\right)+\sigma \xi_{R}\left(t\right) \end{array}$$

describe the temporal dynamics of the firing rates (*r_L_, r_R_*) for each of the two populations, and may be interpreted as originating from a neural network as shown in Figure 4A. Each pool has recurrent excitation (ω_+_), and mutual inhibition (ω_−_). Although the schematic indicates that both excitation and inhibition emanate from a single population of excitatory neurons, this connectivity could be achieved with an equivalent network of excitatory and inhibitory subpopulations (Wong and Wang, 2006; Moreno-Bote et al., 2007; Wong et al., 2007; Marcos et al., 2013; Thura et al., 2022). In particular, we refer to the work by Wong and Wang (2006) in which they reduced a spiking neural network of both excitatory and inhibitory neurons to a two-variable system describing the firing rate of the mean-field dynamics of two populations of excitatory neurons. We opted for this simplified architecture because it is equivalent to the more complex model under certain conditions and provides a more compact formulation. Furthermore, the network shares a basic feature with many other models of bi-stability: to ensure that only one population is active at a time (mutual exclusivity; (Leopold and Logothetis, 1999; Rubin, 2003)), mutual inhibition is exerted between the two populations (Blake, 1989; Laing and Chow, 2002; Wilson, 2003). The overall neuronal dynamics are regulated by the time constant τ, and Gaussian noise ξ with zero mean and standard deviation σ. The sigmoidal function *f* is defined as $f\left(x\right)=F_{\max}/\left(1+\exp\left(-\left(x-\theta \right)/\tilde{k}\right)\right)$ , with $F_{\max}$ denoting the firing rate saturation value.

The neural dynamics described in this section refer to the time-course of a single trial, and are related to the discrimination of the two stimuli. The model commits to a perceptual decision when the difference between the L and R pool activity crosses a threshold Δ (Roxin and Ledberg, 2008), see Figure 4B. This event defines the trial’s decision time. Note that the decision time and the likelihood of picking the larger stimulus are conditioned on the evidence associated with the two stimuli (λ_L_, λ_R_), i.e., how easy it is to distinguish between them. The larger the difference between the stimuli, the more likely, and quickly, the larger stimulus is selected.

This type of decision-making model is made such that the larger stimulus is always favored. Indeed, according to Eq. 1, the target with the stronger evidence is the most likely to be selected. As described in the next section, the addition of the middle layer of our model provides a generalization of this mechanism by allowing the choice between the smaller and the larger target.

#### 2.3.2 Layer 2: Intended decision

While most decision-making models consider only one-shot decisions (Wong and Wang, 2006; Roxin and Ledberg, 2008; Salinas, 2008; Hernández et al., 2010; Kilpatrick et al., 2019), the increased temporal span and the various sources of uncertainty inherent in the consequential task necessitate the addition of a layer to the model. The second layer of the model enables dynamic shifting between the natural impulse to choose the larger stimulus and inhibition. We implemented such a mechanism by means of an inhibitory control pool, which regulates the reversal of the selection criterion toward the smaller or larger stimulus. We called this mechanism *intended decision*, as it defines the intended target to select at each trial. This layer enables the model to switch preference as a function of context (see layer 3 description).

**Figure 5:**
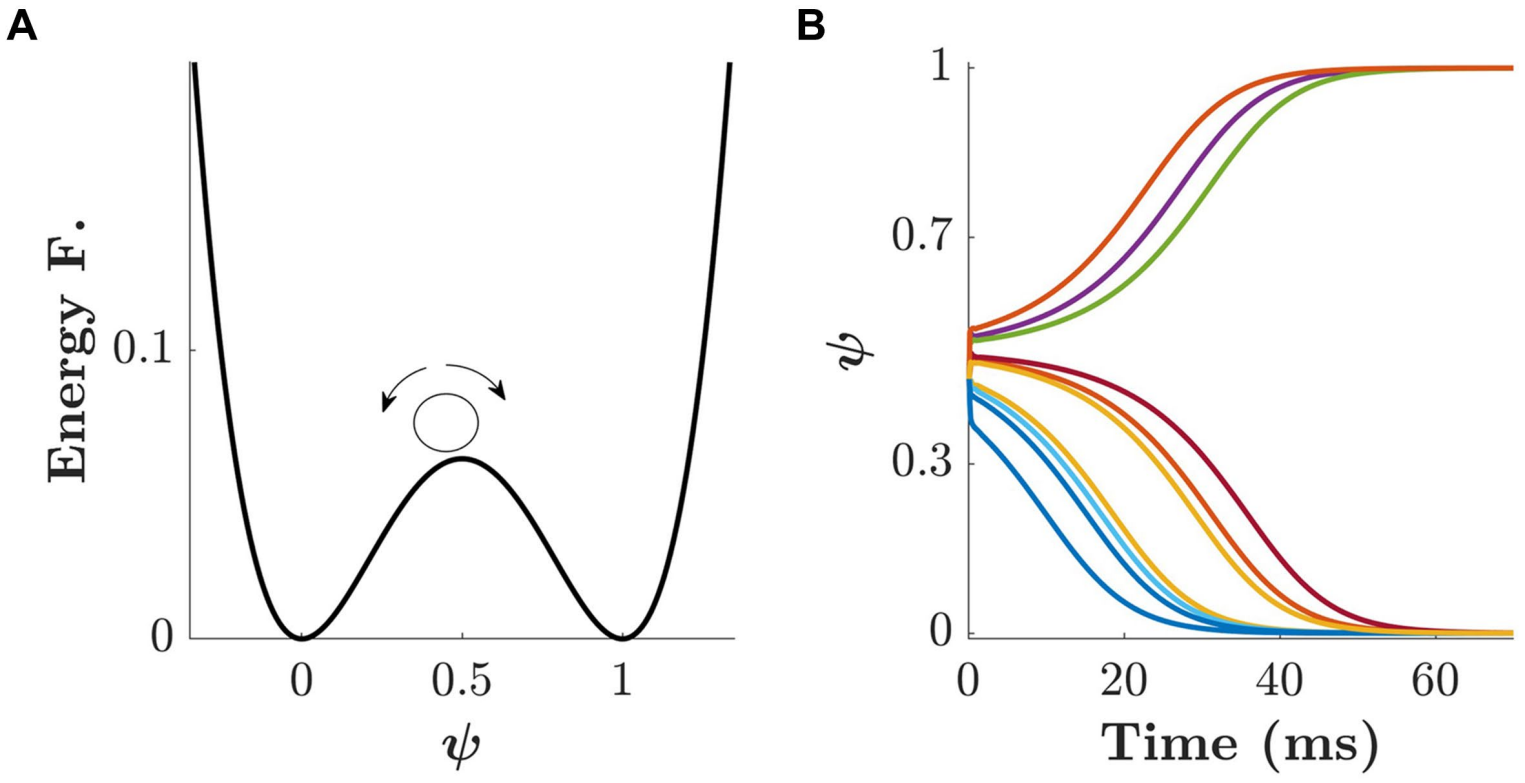
Dynamics of the second layer of the model. **(A)** Energy function $E\left(\psi \right)=\psi^{2}{\left(\psi -1\right)}^{2}$ with two basins of attraction at 0 and 1, associated with the small/big targets, respectively. The small circle represents a possible initial condition for the dynamics of ψ . **(B)** Ten simulated trajectories for ψ(t) according to Eq. 2 with initial condition ψ(0)=0.45 and noise amplitude σ_ψ_ = 0.4 ms^−1^.

The intended decision mechanism is represented by a two-attractor dynamical system. The state of the model can be interpreted as the continuous expression of the tendency to select one choice over another. The attractors are the states toward which the dynamics of the system naturally evolve. Since we have two choices, we considered the energy function $E\left(\psi \right)=\psi^{2}{\left(\psi -1\right)}^{2}$ which has two basins of attraction at 0 and 1. The basins at 0 and 1 are associated with the small and big stimulus, respectively (see Figure 5A). Hence, the dynamics of *ψ* are determined by


(2) $$\tau_{\psi }\frac{d\psi \left(t\right)}{dt}=-4\psi \left(t\right)\left(\psi \left(t\right)-1\right)\left(\psi \left(t\right)-1/2\right)+\frac{1}{t^{2}}\sigma_{\psi }\xi_{\psi }\left(t\right)$$

where τ*_ψ_* is a time constant. The Gaussian noise *ξ_ψ_(t)* is scaled by a constant (*σ_ψ_*) and decays quadratically with time. Thus, the noise exerts a strong influence at the beginning of the process and becomes increasingly negligible as the system approaches either of the basins.

If we set the initial condition to $\psi_{0}=0.5$ and let the system evolve, the final state would be either 0 or 1 with equal probability. Shifting the initial condition toward one of the attractors results in an increased probability of the system ending in the corresponding basin, and ultimately its fixed point. Figure 5B shows 10 simulated trajectories of ψ(t) where the initial condition was set to $\psi_{0}=0.45$ . Since the initial condition is smaller than 0.5, most of the trajectories reach the fixed point at 0 and only a few of them, due to the initial noise, reach 1 as their final state.

The initial condition ( $\psi_{0}$ ) and the noise intensity (*σ_ψ_*) are interdependent. The closer an initial condition is to one of the attractors, the larger the noise must be to escape the corresponding basin of attraction. Behaviorally, the role of the initial condition is to capture the *a-priori* bias of choosing the smaller/bigger target. Please note, however, that a strong initial bias toward one of the targets does not guarantee the final decision, especially when the level of uncertainty is large. Because of this behavioral effect, we refer to the noise intensity *σ_ψ_* as *decisional uncertainty.*

The evolution of the dynamical system in Eq. 2 describes the intention of the decision-making process, at each trial *T*, to choose the smaller/bigger target. The intention is established once a fixed point is reached. We call $\tilde{\psi }\left(T\right)$ the fixed point reached at trial *T*, i.e.,


$$\tilde{\psi }\left(T\right)=\underset{t\to \infty }{\lim}\psi \left(t\right)=\{\begin{array}{c} 0\\ 1 \end{array}$$

is the intended decision of choosing the smaller (0) or bigger (1) stimulus.

Although the small/big stimulus may be favored at each trial, the final decision still depends on the stimuli intensity ratio. More specifically, if the evidence associated with the small/large stimulus is higher/lower than that of its counterpart, the dynamics of the system will evolve as described in the previous section, see Eq. 1. For this reason, we incorporated the *intention* term $\tilde{\psi }\left(T\right)$ in Eq. 1 which connects the *intended decision layer* with the *neural dynamics layer*. This yields a novel set of equations


(3) $$\left\{\begin{array}{c} \tau \frac{dr_{L}\left(t\right)}{dt}=-r_{L}\left(t\right)+f\left(\begin{array}{l} \tilde{\psi }\left(T\right)\lambda_{L}+\left(1-\tilde{\psi }\left(T\right)\right)\lambda_{R}\\ +\omega_{+}r_{L}\left(t\right)-\omega_{-}r_{R}\left(t\right) \end{array}\right)+\sigma \xi_{L}\left(t\right)\\ \tau \frac{dr_{R}\left(t\right)}{dt}=-r_{R}\left(t\right)+f\left(\begin{array}{l} \tilde{\psi }\left(T\right)\lambda_{R}+\left(1-\tilde{\psi }\left(T\right)\right)\lambda_{L}\\ +\omega_{+}r_{R}\left(t\right)-\omega_{-}r_{L}\left(t\right) \end{array}\right)+\sigma \xi_{R}\left(t\right) \end{array}\right.$$

which is able to switch preferences between the large and small stimulus. If $\tilde{\psi }\left(T\right)=1$ , the larger stimulus is favored (and the equations reduce to Eq. 1); however, if $\tilde{\psi }\left(T\right)=0$ the smaller stimulus is preferred.

In summary, the *intended decision* layer enables the model to dynamically adjust preferences for the bigger or smaller stimulus. This inhibitory control plays the role of the regulatory criterion (size-wise) with which a decision is made in the consequential task, as described by Eq. 2.

#### 2.3.3 Layer 3: Learning the strategy

Although the previously described intended decision layer enabled the model to target a specific type of stimulus at each trial, a second mechanism is required to internally oversee performance and to promote beneficial strategies. In the consequential task, the goal is to maximize the cumulative reward value obtained in each episode. As shown in previous analyses, most participants learned the optimal strategy after an exploratory phase, gradually improving their performance until the optimum was reached. Inspired by the same principle of exploration and reinforcement, we incorporated the strategy learning layer in our model.

The internal dynamics of an episode are such that selecting the small/large stimulus in a trial results in an increase/decrease of the mean value of the presented stimuli in the next trial (Figure 1). Consequently, the strategy to maximize the reward value must vary as a function of trial within episode (*T_E_*). For clarity, each trial *T* is associated with an episode *E* and number of trial within episode *T_E_*. We use both notations interchangeably, i.e., *T =* (*E, T_E_*).

The strategy learning mechanism in the model reinforces beneficial strategies and weakens less rewarding ones, see Discussion for a comparison with existing models. Following each episode *E,* the strategy function $\phi =\phi \left(E,T_{E}\right)$ is updated by considering the intended choice $\tilde{\psi }\left(T\right)$ and the obtained reward value *R(T)*. In our case, reward value originates from each participant’s subjective evaluation in the absence of explicit performance feedback. This internal assessment yields a positive or negative perception of reward, i.e., a subjective reward. Learning implies that the preference for the selected strategy is reinforced if the participant’s internal assessment results in positive subjective reward. Namely, with a positive reward (*R(T) > 0*), ϕ is increased if the larger stimulus was chosen ( $\tilde{\psi }\left(T\right)=1$ ) and decreased otherwise ( $\tilde{\psi }\left(T\right)=0$ ). Notice that a negative reward discourages the current strategy but promotes the exploration of alternative strategies and makes it possible to learn the optimal one over time. Mathematically, we describe the dynamics of learning as


(4) $$\begin{array}{l} \phi \left(E+1,T_{E}\right)=\phi \left(E,T_{E}\right)\\ +kR\left(E,T_{E}\right)\left(2\tilde{\psi }\left(E,T_{E}\right)-1\right){\left(\phi \left(E,T_{E}\right)-1\right)}^{2}{\left(\phi \left(E,T_{E}\right)\right)}^{2} \end{array}$$

where *k* is the learning rate. Note that if *k = 0*, $\phi \left(E,T_{E}\right)$ remains constant and, therefore, there is no learning. The term ${\left(\phi \left(E,T_{E}\right)-1\right)}^{2}{\left(\phi \left(E,T_{E}\right)\right)}^{2}$ is required to gradually reduce the increment to zero the closer ϕ gets to either zero or one. This bounds ϕ to the interval [0, 1]. The reward function *R(* $E,T_{E}$ *)* represents the subjective reward. The only requirement for this function is that *R*( $E,T_{E}$ ) must be positive or negative if the subjective reward is considered beneficial or not, respectively. In the case of the current task, participants must look for clues that convey indirect information about their performance. The key observation participants had to make was the change in stimuli mean *M* between consecutive trials in an episode as a result of their choices. For this reason, we defined the reward function as $R\left(E,T_{E}\right)=M\left(E,T_{E}+1\right)-M\left(E,T_{E}\right)$ (see Eq. 4). We discuss how the reward function could generalize to different tasks in the conclusions section.

The strategy layer operates a longer time scale than the lower layers. The strategy is updated at the end of each episode by reinforcing/weakening the policy that has yielded a positive/negative reward. Mathematically, as mentioned before, this means that with a positive reward (*R(T) > 0*), ϕ is increased if the larger stimulus was chosen ( $\tilde{\psi }\left(T\right)=1$ ) and decreased otherwise ( $\tilde{\psi }\left(T\right)=0$ ). In the case that both the larger stimulus is repeatedly chosen and positive rewards are obtained, then ϕ converges to 1. In contrast, if both the smaller stimulus is repeatedly chosen and positive rewards are obtained, then ϕ converges to 0. This update manifests as a change in the initial condition for the intended decision ψ (Eq. 2), i.e., biasing the direction, small or big, for the intended decision to go. As shown in Figure 5, shifting the initial condition toward one of the two basins (0 or 1) increases the probability of reaching it. Mathematically, this can be implemented by setting ψ(0)=ϕ(T) for each trial. In this way, the connection between the intended decision and strategy layers lies in the influence the strategy learning exerts at each decision.

**Figure 6:**
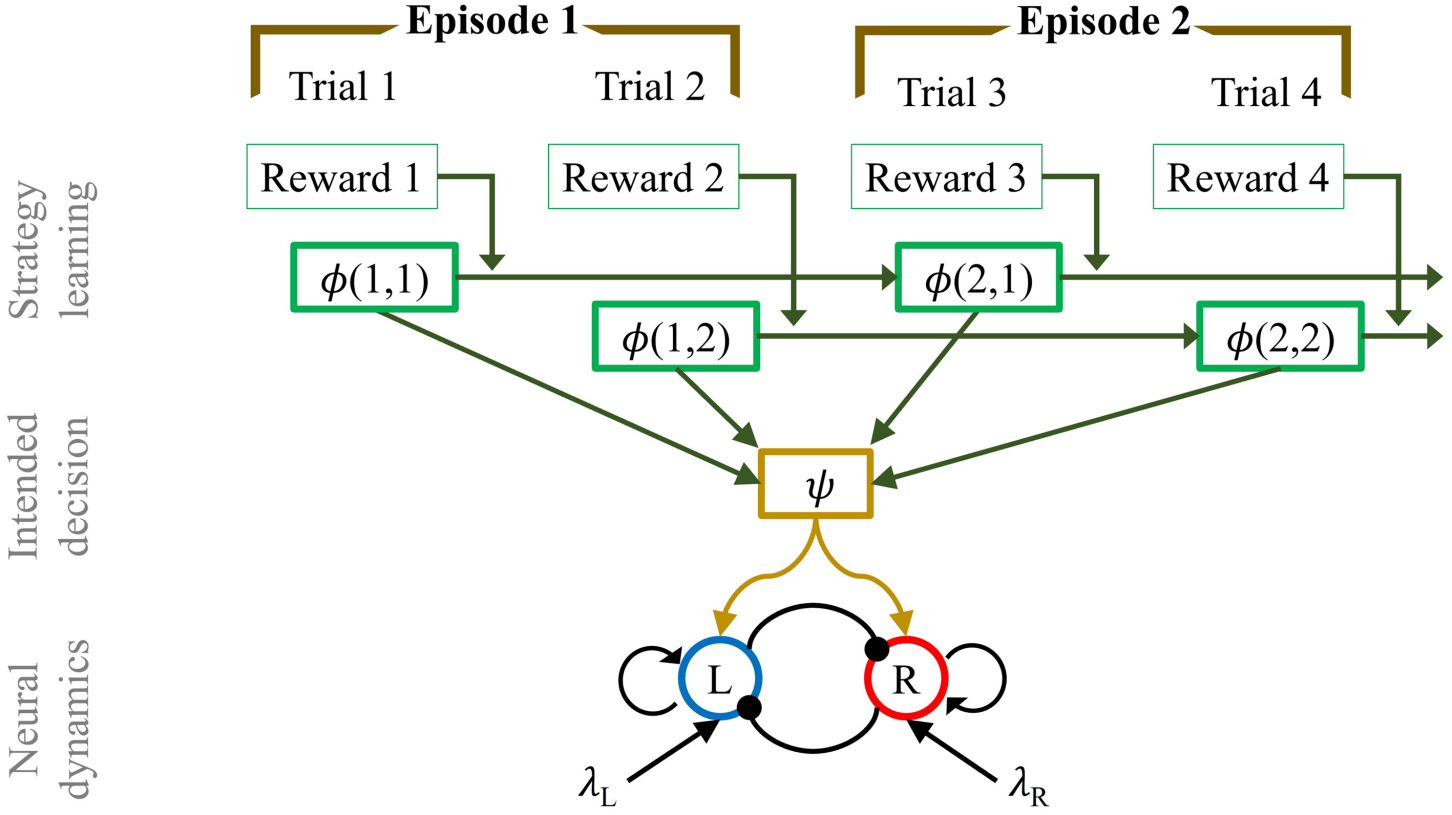
Multi-layer network structure of mean-field model of consequence-based decision making, in the case of a horizon 1 experiment. From the bottom: Neural dynamics layer: pool L is selective for stimulus L (λ_L_), while the other population is sensitive to the appearance of stimulus R (λ_R_). The two pools mutually inhibit each other (ω_−_) and have self-excitatory recurrent connections (ω_+_). The dynamics of the firing rate of the two populations is regulated by Eq. 3. Intended decision layer: the function ψ represents the intention, in terms of decision process, made at each trial T, of aiming for the smaller or bigger target. The dynamics of the intended decision is regulated by Eq. 2. Strategy learning layer: after each trial the strategy is revised, in a reinforcement learning fashion, depending on the magnitude of the gained reward value. The strategy is updated according to Eq. 4.

To conclude, our model consists of a three layer structure. The dynamics of each layer are defined by Eq. 3 (neural dynamics), Eq. 2 (intended decision), and Eq. 4 (strategy learning). Figure 6 shows a schematic of the complete model. The bottom part depicts the neural dynamics originating from two pools of neurons which encode the responses to two external stimuli (*L, R*). The middle shows the intended decision layer at every trial. Finally, the top is the strategy learning layer which evolves at a much slower timescale; the combined information of the intended decision and the subjective reward drives strategy learning.

### 2.4 Model simulations

**Figure 7:**
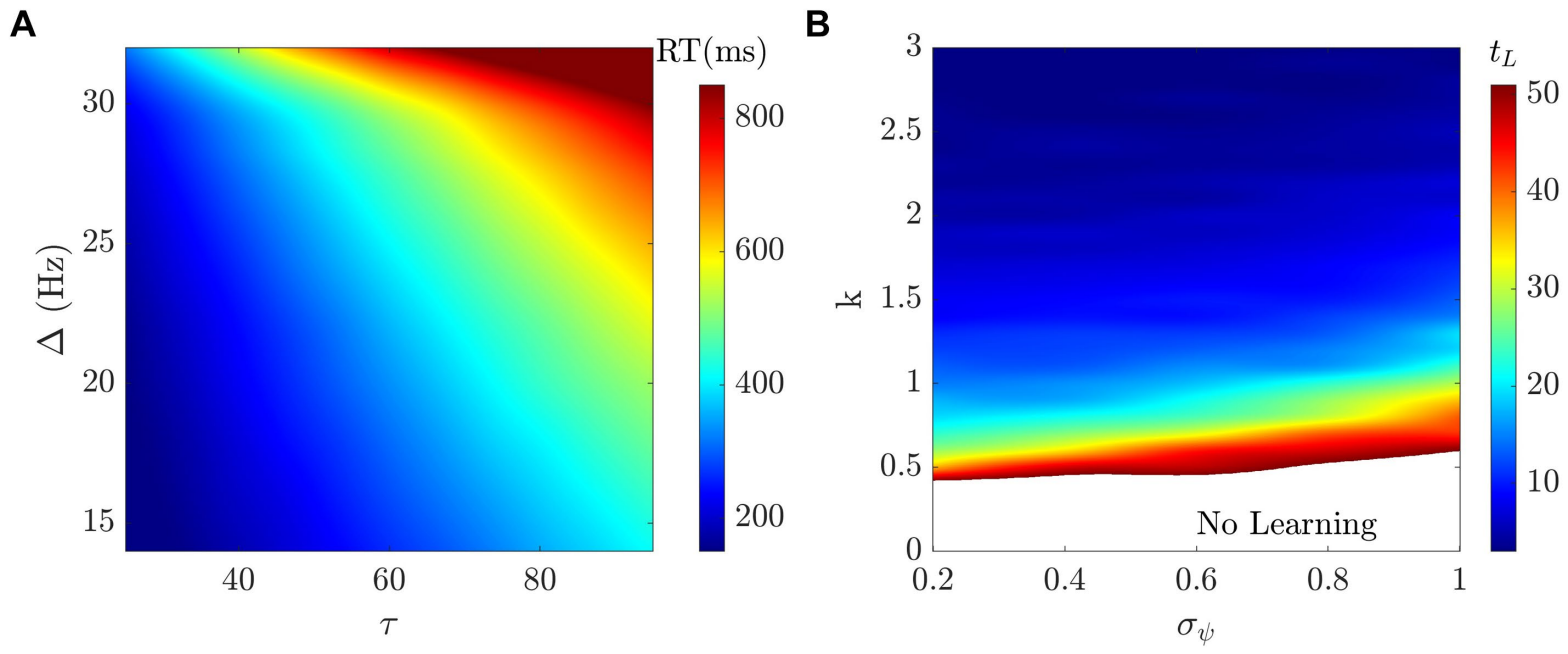
Parameter space analysis. **(A)** The RT increases when increasing either τ or Δ (*σ* = 0.001 ms^−1^). **(B)** Learning time (t_L_) decreases when learning rate k increases and when decisional uncertainty decreases σ_ψ_ (*τ* = 81 ms, σ = 0.001 ms^−1^, and Δ = 30 Hz).

We performed a parameter space analysis to assess the influence of the model parameters on the main behavioral metrics of interest: reaction time (RT) and performance (PF). To obtain meaningful biophysical results for the neuronal dynamics, we simulated our model varying the time constant τ, the noise amplitude σ, and the decision threshold Δ (in Eq. 3) in the following ranges: τ∈[25,95] *ms*, $\sigma \in \left[10^{-3},10^{-2}\right]$ *ms^−1^*, and Δ∈[0.01,0.035] *ms^−1^* (see (Marcos et al., 2013). Also, we set F_max_ = 0.04 *ms^−1^*, θ = 0.015 *ms^−1^*, $\tilde{k}$ = 0.022 *ms^−1^*, ω_+_ = 1.4, ω_−_ = 1.5. We fixed the parameters defined in the function *f* (see Eq. 3) as well as the connection strengths between pools of neurons (ω_+_ and ω_−_), as in (Marcos et al., 2013). As we will see below, by only varying τ, σ, and Δ we can simulate a wide range of different behaviors. In Eq. 2, we set τ*_ψ_* = 10 *ms* such that the dynamics of Eq. 2 is faster than the dynamics of Eq. 3 while remaining the same order of magnitude. Figure 7A shows how RT is affected by τ and Δ. By increasing the time constant τ, the RT increases both in mean and standard deviation (see Supplementary Figures S4a,d). The same trend occurs when increasing the threshold Δ (Supplementary Figures S4b,e). When varying the noise σ, we did not find a substantial difference in the RT (Supplementary Figures S4c,f). By fixing τ, σ, and Δ, we quantified the influence of the learning rate *k* and the decisional uncertainty *σ_ψ_* on the PF, and, consequently, on the learning time *t_L_* (defined as in section Behavioral Results). Figure 7B shows that learning time decreases as learning rate *k* increases and decisional uncertainty *σ_ψ_* decreases. Note that for these simulations we used *n_H_ = 1* with 50 episodes, therefore any *t_L_* bigger than 50 means the optimal strategy was not learned.

**Figure 8:**
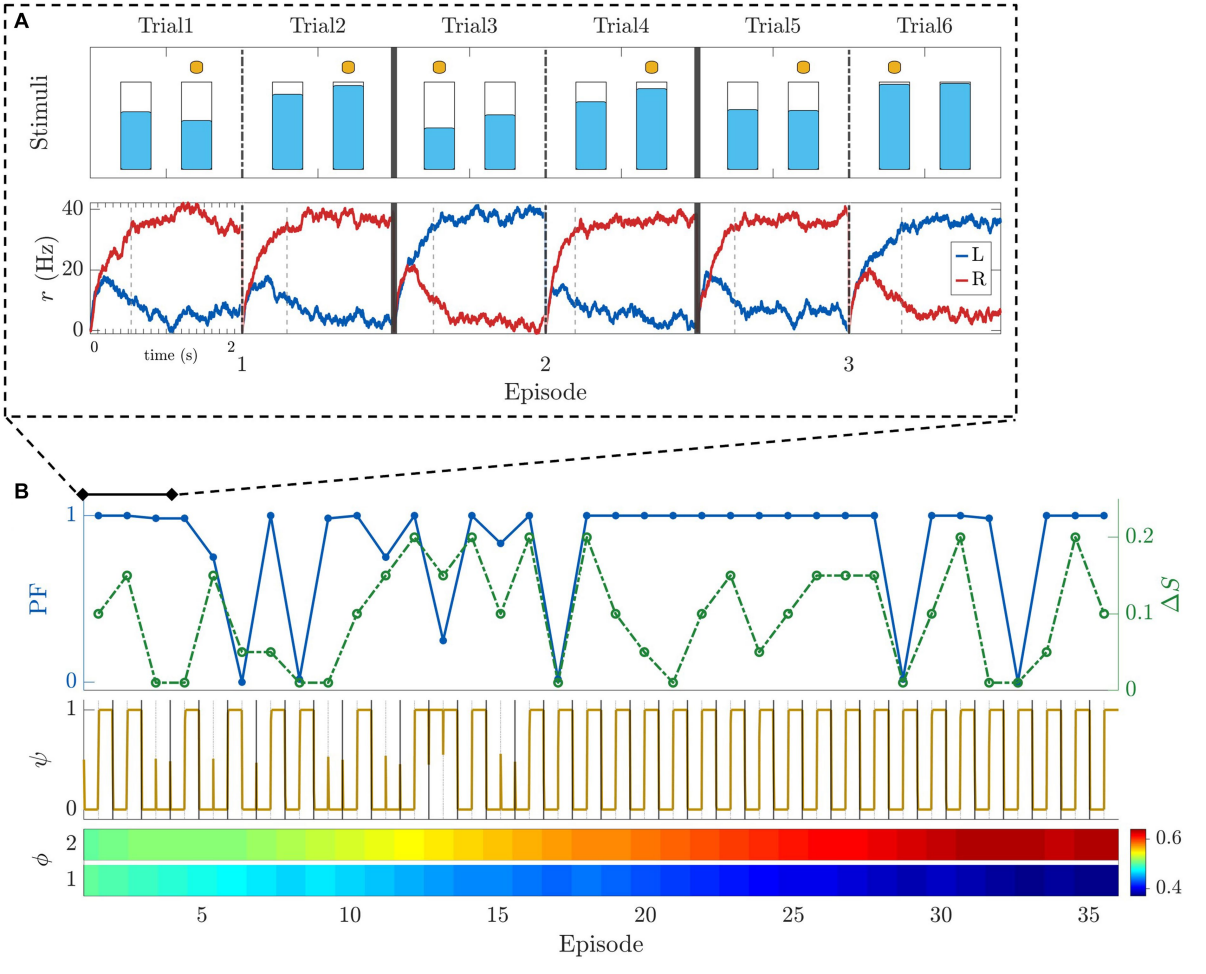
Model example simulations for a horizon 1 block. **(A)** Simulation of the first 3 episodes. Top row: Stimuli presentation with selections indicated by a yellow dot. Bottom row: firing rate of the two populations of neurons encoding the left (in blue) and right (in red) stimuli (Eq. 3). Vertical dashed bars indicate the time the decision threshold was crossed. **(B)** Simulation of 36 consecutive episodes. First row: Performance (blue - solid) and difference between stimuli ΔS (green - dashed). Second row: intended decision dynamics of choosing the bigger (1) or smaller (0) stimulus. Third row: evolution of strategy learning for each trial within episode (*T*_*E*_). Parameters used for the simulations: *G* = 0.3, Δ = 25 Hz, *τ* = 80 ms, *σ* = 0.006 ms^−1^, $\phi_{0}\left(1,T_{E}\right)=0.5$ for *T*_*E*_ = 1,2, *k* = 0.4, σ_ψ_ = 0.4 ms^−1^.

To demonstrate the behavior of the model, Figure 8 shows the results of a typical simulation of a horizon *n_H_ = 1* experiment. Figure 8A shows the dynamics of the neural dynamics layer of our model together with the stimuli used in the simulation during the first three episodes. The bottom row shows the time course of the two population firing rates (Eq. 3) encoding the stimuli L, R (depicted in the top row). To better understand the progression of this process over time, Figure 8B provides a view of 36 episodes. The top row shows the performance and difficulty (in terms of difference between stimuli ΔS) metrics. Note that the optimal strategy in this simulation was learned and applied from the 17^th^ episode onward. After this point, only the most difficult trials (smallest ΔS) managed to diminish the performance. The same conclusions can be drawn by looking at the time course of the intended decision metric (middle inset). After the 17th episode the intended decision metric exhibits a repeating pattern (small for *T_E_ = 1*, and big for *T_E_ = 2*). The bottom row shows the strategy learning. For the first trial within episode (*T_E_ = 1*), *ϕ* tends to 0, i.e., it pushes the intended decision to choose the smaller stimulus. For the second trial within episode (*T_E_ = 2*), the trend is reversed, effectively capturing the optimal policy.

### 2.5 Individual participants’ behavioral fit

In this section we describe the fit of the model parameters to the participants’ individual behavioral metrics. The first step is to find the best fit for the neural dynamics by fitting the reaction time (RT) and the visual discrimination (VD), i.e., fit the parameters involved in Eq. 3. These parameters have a biological meaning, and therefore they should be fit to the corresponding measures in the neural data. However, in our case we are only aiming to fit behavioral data. As shown in Figure 7, and discussed in the corresponding section, *σ* does not have an influence on RT, and the same mean RT can be found for different combinations of τ and Δ. In the absence of neural data, it would be meaningless to fit all parameters to RT since this would lead to overfitting. Therefore, in order to reduce the number of parameters to fit, we fix *σ = 0.001 ms^−1^,* and we vary τ and Δ dependently to explore the parameter space unidimensionally. Specifically, we vary τ∈[25,95] *ms* and $\Delta =2.57\cdot 10^{-4}\tau +0.0076$ *ms^−1^*, which corresponds to the diagonal in Figure 7A.

The remaining steps of the fitting process pertain to the behavioral metrics. The second step consists of calculating the initial preferential bias *ϕ_0_*. Finally, in the third step, we run the model using the previously established parameters to find the best fit for *σ_ψ_* and *k*, i.e., the decisional uncertainty and the learning rate. Following the same argument as before, we reduced the number of parameters to fit. Since the same mean learning time can be obtained for different combinations of *σ_ψ_* and *k*, as shown in Figure 7B, we fix *σ_ψ_* = *0.6* and vary k∈[0,2.5] .

To test the robustness of the fitting method, and to test whether we are overfitting, we performed a parameter recovery analysis (White et al., 2018; Evans et al., 2020; Danwitz et al., 2022). We obtained correlation coefficients close to 1, which reflect an excellent recovery, see Supplementary Figure S5.

We fit the parameters in a sequential fashion because the estimates of both RT and VD depend uniquely on Eq. 3. In order to evaluate the dynamics of the perceptual processes, RT and VD are fit using horizon *n_H_ = 0* only. Once these have been established, we focus on the behavioral part, by fitting the initial preferential bias and the learning rate for different horizons.

#### 2.5.1 Reaction times and visual discrimination

The first metric to fit is each participant’s RT. As explained above, to perform this fit we use Eq. 3 and data from *n_H_ = 0*, by varying τ∈[25,95] *ms.* Note that due to response anticipation of the GO signal, the experimental RTs could be negative in a few cases (see Figure 3C). A free parameter was incorporated into the model to control for this temporal shift.

The second metric to fit is the VD, i.e., the ability to distinguish between stimuli. We assumed VD to be specific to each participant, and constant across blocks of each session. As a means of assessment, we checked how often the larger stimulus had been selected over the last 80 correct trials of the *n_H_ = 0* block for each level of difficulty. The only case where accuracy was low was the highest difficulty level (ΔS = *0.01*). For our model to capture this, we used a linear transformation $\tilde{s}=\alpha +\beta s$ to re-scale the stimuli *s*, ranging from 0 (empty) to 1 (full), to a range more meaningful for the model ( $\lambda_{L,R}\sim 10^{-2}$ , (Moreno-Bote et al., 2007)). Additional constraints were set for α and *β* so that this transformation would not swap the intensities between stimuli (i.e., if $s_{L}\geq s_{R}$ then ${\tilde{s}}_{L}\geq {\tilde{s}}_{R}$ ), and so that the input stimuli would always be positive ( ${\tilde{s}}_{L,R}>0$ ). Abiding by these conditions, we varied *α* and *β* and ran a grid-search set of simulations of Eq. 3 (with $\Delta S=\left|s_{L}-s_{R}\right|=0.01$ ). We calculated how often the firing rate of the population encoding the larger stimulus was bigger than the alternative. The result depends not only on α and *β*, but also on *τ*, *σ*, and Δ. Thus, to capture the large variety of results encompassed by the ranges of *τ*, *σ*, and Δ, while abiding by the aforementioned constraints, we fix *α* = −0.018, and let *β* vary between 0 and 0.1. These conditions allowed for proper exploration of the parameter space.

**Figure 9:**
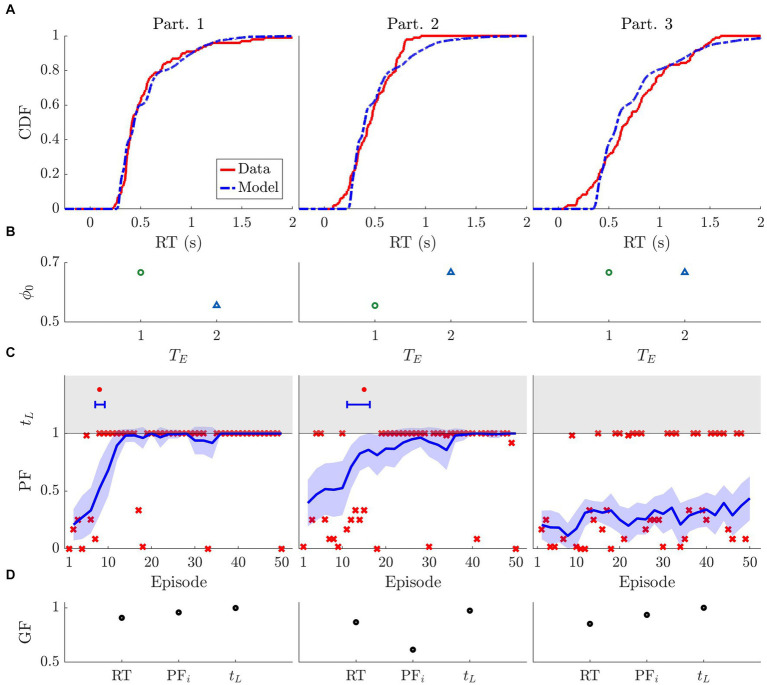
Model fit to three sample participants’ behavioral metrics. Data used: one block of horizon 1. The specific parameter values of the fit are displayed in Table 1. **(A)** Cumulative distribution function (CDF) of the reaction times (RT) for the participant data (solid red) and model simulation (dashed blue). **(B)** Initial bias ϕ_0_ of the participant at the beginning of the block for each trial within episode (*T*_*E*_). The more the preferred choice tends toward choosing the larger (smaller) stimulus, the bigger (smaller) ϕ_0_ is. **(C)** Bottom: Performance of the participant (red crosses) and of the model’s simulations (blue line: mean, shaded area: confidence interval). Top: Learning time for the participant (red dot) and model simulations (blue error bar). **(D)** Goodness of fit (GF) for three metrics: reaction time (RT), initial performance (PF_i_), and learning time (t_L_). Goodness of fit is calculated as follows: RT = 1-Kolmogorov–Smirnov distance between CDF, PF_i_ = 1- mean square error, t_L_: 1- difference between learning times of participant and model’s mean divided by the total number of episodes.

We ran 100-trial simulations of a horizon *n_H_ = 0* block for each combination of the parameters *τ* and *β*. We then calculated the empirical cumulative distribution functions (CDF) of the RTs for all trials, and the VDs only for the difficult trials, i.e., when ΔS = *0.01*. The distribution of simulated RTs was then compared to the distributions of experimental RTs by means of the Kolmogorov–Smirnov distance (KSD) between CDFs (Smirnov, 1948; Stephens, 1974; Quinn and Keough, 2002; Marsaglia et al., 2003). Since both RTs and VDs strongly depend on the parameters, both were fit simultaneously. Namely, we consider the error metric $\hat{M}=KSD+c\;|VD^{sim}-VD^{real}|$ , with *c* being a constant set to 0.4 to balance the weight of the two metrics, and VD^sim^, VD^real^ being the VD from the simulated and real data, respectively. The parameters τ and β that minimize $\hat{M}$ are selected for the fit. Figure 9A depicts the CDF of the RT for the participants and for the best-fit model simulation.

To summarize, in the first step of the fit, we focused on the neural dynamics layer by fitting all the free parameters of Eq. 3, i.e., *τ* and *β*, corresponding to RT and VD. The subsequent steps consider the behavioral component of the data.

#### 2.5.2 Initial preferential bias

Each participant performing the task might have an initial choice preference, i.e., a natural bias toward the larger (or smaller) stimulus. In our model this is captured by the parameter *ϕ_0_* in Eq. 4. In the absence of bias, *ϕ_0_* equals 0.5. The greater the preference toward the bigger choice, the closer to 1 *ϕ_0_* will be.

We set a vector of initial conditions $\phi \left(E=1,T_{E}\right)=\phi_{0}\left(T_{E}\right)$ for each trial within episode *T_E_*. To quantify *ϕ_0_*, we selected the first 3 episodes for each participant, and calculated the frequency *f* with which the larger stimulus was selected. The parameter *ϕ_0_* functions as an initial condition for the intended decision process (see Eq. 2). In agreement with the attractor dynamics, if the initial condition coincides with one of the basins of attraction, the system will be locked in that state. To prevent this (since *ϕ_0_* should only be an initial bias), we rescaled the frequency of the selected choices *f* to make the value closer to 0.5, i.e., $\phi_{0}=\left(1+f\right)/3$ (other rescaling factors could be used and would not change the results). Figure 9B shows the values obtained for *ϕ_0_* for each trial within episode *T_E_*. Note that we have selected one block from *n_H_ = 2* for participant 2 and *n_H_ = 1* for the others.

#### 2.5.3 Learning rate

Finally, to fit the remaining parameter *k* to each participant’s data, we ran the model using the previously established parameters (τ, β, and *ϕ_0_*) and fit the resulting performance to that of each participant. For each *k*, we ran 50 simulations and extracted the performance mean and standard deviation. To compare model and participant performances, we considered different metrics such as maximum likelihood, Bayesian (BIC) and Akaike information criterions (AIC) (Smirnov, 1948; Stephens, 1974; Huber-Carol et al., 2002, 2017; Nikulin and Chimitova, 2017). While these are common metrics for model comparison, they disregard the specific time dependency throughout each block, which is a key factor to characterize the learning process of the participant. Classical maximum likelihood, for example, would be strongly affected by trials exhibiting low performance due to participant fatigue or distraction. This renders the metric unsuitable for our purpose. Recently, more complex methods have been developed to overcome this issue, such as in (Boelts et al., 2022). Nevertheless, we do not require such complex metrics since our goal is to show that the model can fit the full range of the participants’ data, not to compare goodness of fit to other models. To this end, we designed an *ad-hoc* metric consisting of two components to determine goodness of fit. The first component is the initial condition, obtained by calculating the mean-square error of the performance between the model and the data during the first five episodes. By minimizing the mean-square error, we ensured that the learning process began under similar conditions for the model and for the participant. The second factor is the time required to learn the strategy. As already outlined in the Behavioral Results section, we defined the time at which the strategy was learned as the moment after which the optimal strategy was employed in at least 9 out of the following 10 episodes, and 75% of the remaining episodes until the end of the block. To ensure that a low success rate was not due to errors caused by visual discrimination, we excluded the episodes with ΔS *= 0.01* from this part of the fit. In summary, by combining the results for the initial conditions (*I*) and the learning time (*L*), we could extrapolate the best fit for *k* by minimizing the linear combination L+0.1.I .

Figure 9C shows the participants’ performance (red marks) as well as the associated best-fit model performance (the blue line is the mean, and the colored area is the 95% confidence interval). The top part of the plots depicts the learning time (*t_L_*) calculated for the participant (red mark) as well as for the best fit model simulations (blue error-bar). Table 1 shows the best-fit parameter values per participant.

**Table 1:** Parameter values obtained when fitting data from 1 block for each of the 3 participants.

P.	*GF (RT, PF_i_, t_L_)*	*t_L_*	*k*	*τ*	*β*	*ϕ_0_* (*T_E_*)
1	{0.91,0.96,1}	8	2.8	67	0.057	{0.67,0.56}
2	{0.87,0.62,0.97}	14	0.5	60	0.051	{0.56,0.67}
3	{0.85,0.93,1}	–	0.4	67	0.045	{0.67,0.67}

The parameters *τ* and *β* refer to Eq. 3; *ϕ*_0_ and *k* belong to Eq. 4. The learning time (t_L_) and the goodness of fit (GF) are shown in the first 2 columns.

All participants except one learned the strategy yielding maximum reward value. Participant 1 learned very quickly (in just 8 episodes). The model fit to participant 1 yielded the highest learning rate (*k = 2.6*). Interestingly, even though participant 3 did not learn the correct strategy, the parameters obtained from the fit still indicated some learning (*k = 0.2*). Note that, though participant 2 learned the strategy fairly quickly (after only 15 episodes), participant 2’s learning rate was only slightly greater than participant 3’s despite participant 3 never learning the optimal strategy. The reason the learning rates for these two participants are similar, even though they reflect two distinct strategies, lies in the initial condition. Namely, participant 3 began the task with a stronger bias toward choosing the larger stimulus ( $\phi_{0}\left(T_{E}\right)=\left\{0.67,0.67\right\}$ vs. {0.56,0.67} for participant 2). Such disadvantageous initial conditions combined with a weak learning rate was not enough for the strategy to be learned in a block of 50 episodes.

Figure 9D shows the goodness of fit for the two main behavioral metrics we aimed to reproduce: the reaction time (RT) and the performance in terms of initial performance (*PF_i_*) and learning time (*t_L_*). To measure the goodness of fit while remaining consistent with our fitting procedure, we used the following metrics: KSD for RT, mean-square error for *PF_i_*, and the difference between the participant’s data and the model’s mean divided by the total number of episodes for *t_L_*.

To summarize, we first found the best fit for the RT and VD by varying τ and β in Eq. 3. Then, we calculated the subjective initial bias *ϕ_0_*. Finally, while holding the aforementioned parameters fixed, we found the best fit for the learning rate *k*.

**Figure 10:**
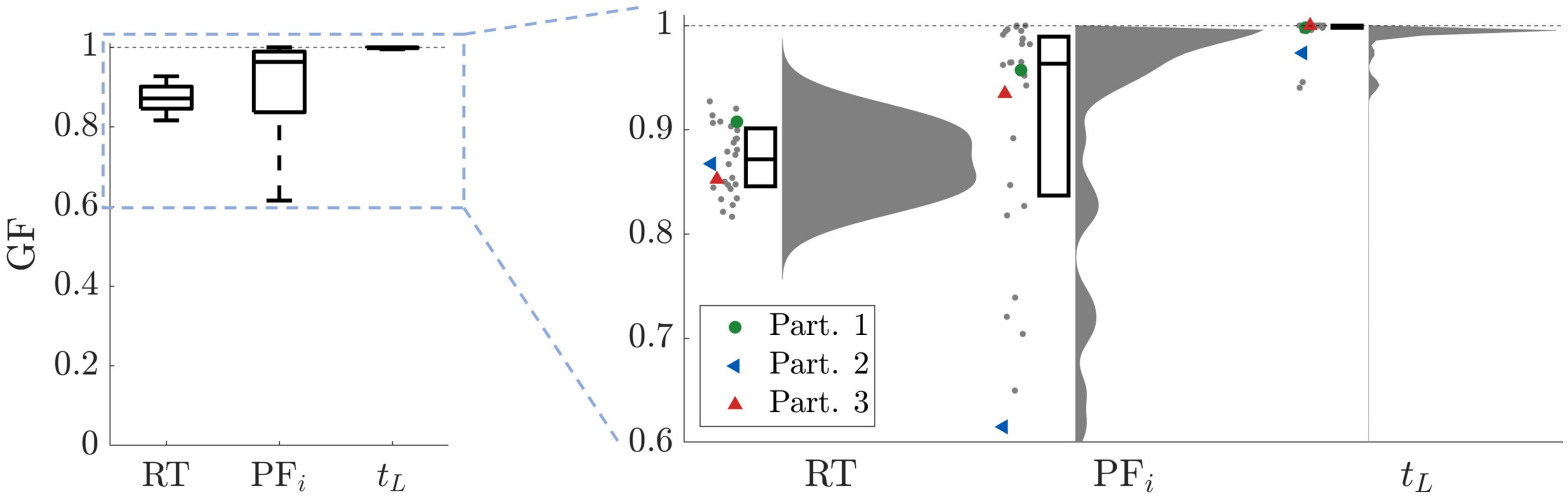
Goodness of fit. For RT we calculated KSD, for PF_i_ we evaluated the mean-square error, and for t_L_ we took the difference between the participant’s data and the model’s mean divided by the total number of episodes. For all three metrics, we show the scatter plot of each single participant, the corresponding distribution, and the boxplot depicting the median and the 25/75 percentiles. For reference, the superposed colored markers indicate the three participants shown in the previous figure.

To illustrate that the model can capture the full range of behavior, Figure 10 shows the goodness of fit for the RT, initial performance PF_i_, and learning time t_L_ for all 28 participants. For all three metrics, we show the scatter plot including each participant, the respective distribution, and the boxplot depicting the median and the 25th/75th percentiles. For reference, we superposed colored markers to indicate three sample participants shown in the previous figure.

In summary, we fit the model to each of the participant’s behavioral metrics. We first used the RT distribution and VD of each participant to fit the parameters in Eq. 3. Once these parameters were fixed, we moved on to calculate the initial bias before running simulations of the model. Finally, we compared the results of the simulations with the performance of the participants and found the best fit for the behavioral parameters, i.e., the learning rate and decisional uncertainty.

## 3 Discussion

In this study we analyzed how the consideration of consequence influences learning in value-based decision-making, and provided an account of the underlying neural processes. To this end, we examined how human participants learned to make sequences of decisions between value-based stimuli in the consequential task. This is a novel experimental task in which initial knowledge about environment was minimal, and explicit performance cues were absent. Consequence refers to the effect choices exert on the value of stimuli in the next trial. This was designed to promote small value choices during the early trials of each episode, and a large value one in the last trial. The instruction to each participant was to explore and to find the strategy leading to the highest cumulative reward value. The absence of explicit performance cues was meant to promote the development of a subjective assessment of performance based on relating the size of the stimuli in the current trial to the choice in the previous one. Our results show that decisions involving the computation of future consequence took longer to perform than those with no further consequence (i.e., the last choice of each episode), suggesting a more involved decision-making process when future consequence is to taken into account. Most participants eventually learned the optimal strategy, although with significant differences in their learning times.

Based on these observations and on previous evidence, we introduced a mathematical model of a set of plausible cognitive processes for consequence-based decision-making. The model is organized in three layers. The bottom layer describes the average dynamics of two neural populations representing the preference for each option. The populations compete against each other until their difference in activity crosses a threshold. The middle layer illustrates the participant’s preference for choosing the bigger or smaller stimulus at each trial (the so-called intended decision). The top layer describes the strategy learning process which oversees the model’s performance, adapts by reinforcement to maximize the cumulative reward value, and drives the intended decision layer. This oversight mechanism, combined with the modulation of preference, accurately reproduced an internal process of consequence assessment and subsequent policy update. The model was validated by fitting its parameters to reproduce each participant’s behavioral data (i.e., reaction time distribution, visual discrimination, initial bias, and performance). The model faithfully reproduced the participants’ behavior despite its varied nature. Importantly, this model also provides a plausible account of the neural processes required for gauging options as a function of their associated consequence (measured in terms of reward), and of how these processes are involved in decision-making.

### 3.1 Justification of the consequential task

Real world decisions are rarely accompanied by immediate feedback, there is often a conflict between short and long-term reward, consequences are often long-lasting, reward is often difficult to quantify, and state-action spaces often require exploration to define (as opposed to being known *a priori*). Several of these characteristics generate uncertainty and complicate performance assessment. The consequential task combined features common to both hierarchical decision-making (Lorteije et al., 2015; Zylberberg et al., 2017; Zylberberg, 2022) and delay discounting paradigms (Hayden and Platt, 2007; Kim et al., 2008; Hwang et al., 2009; Alexander and Brown, 2010; Hayden, 2016) to examine how this kind of decision-making unfolds. Moreover, the absence of cued performance feedback during the task made our paradigm particularly suitable for studying how learning optimal strategies may extend from immediate perceptual decision-making to a more complex process involving predictions of future states. Unlike standard hierarchical decision-making and partially observable Markov decision processes (Smallwood and Sondik, 1973; Kaelbling et al., 1998), participants in the consequential task were not aware of the underlying relationship between actions and their consequences. Participants were told only that the choice they made in one trial might influence the next. In this way, participants had to explore and observe the consequences of their choices to deduce that an inter-trial dependence existed. Moreover, participants could never be certain if they found the optimal solution, i.e., picked the correct sequence of decisions to maximize cumulative reward value. This is in sharp contrast to delay discounting tasks which largely focus on the principle of inhibitory short-term control where the presence of explicit cues helps overcome impulsive behavior, such as in the *farming on Mars* task (Gureckis and Love, 2009) (see below).

### 3.2 Cognitive hypothesis based on behavioral results

The purpose of our study was to understand how participants learned how their choices influenced the decision context, as opposed to assessing whether reward value varied with time. In other words, the absence of explicit cues was intended to force the participants to rely on their own subjective assessment to infer the delayed consequence of their decisions across groups of successive trials, and whether their strategy was being successful. This inner assessment had to be driven by the participant’s probing of patterns of action/decision effects. Complementary to this, we believe that participants had to go through a hypothesis testing process, until the eureka moment of realizing that one specific strategy was better than the others. Consequently, to find the optimal strategy, participants had to first realize that choosing the smaller option lead to more rewarding options (the eureka moment). Explicitly, this implies identifying the specific feature of the stimuli to be considered, having nothing else than the observance of their choice/action effects on the environment (the stimuli in the next trial). Then, they had to confirm their criterion based on the global effect of their choices on the stimuli size across episodes.

### 3.3 Rule-based vs. Far-sighted assessment of consequence

The strategy to attain the highest possible cumulative reward value may be operationalized as a sequence of decision rules: choose small, then big in horizon 1 episodes; choose small, then small, then big, in horizon 2 episodes. Though we expected the participants’ choices to abide by these rules once the learning was complete and the optimal decision strategy was established, the focus of this study is on how consequence-based assessment forms and influences the learning of that optimal strategy. Because of this, it was crucial that the consequential task were devoid of any cued performance feedback, which could potentially inform the participant of his/her performance after each episode and, ultimately, promote a rule-based strategy.

For the same purpose, and to promote exploration, the participants were left with the uncertainty of neither having a criterion to follow to make decisions nor the knowledge about which aspect of the stimuli to attend to while making decisions. Note that, in addition to the bar heights (proportional to reward value), the stimuli at each trial were presented on the right and left of the screen, they were shown sequentially, randomly alternating their order of presentation across trials. Both the position and order of presentation of the stimuli increased the uncertainty with respect to the relevant stimuli dimensions. Under these conditions, participants had to perceive the relationship between their choices and the values of the stimuli presented in subsequent trials. If noticed, this observation could then be used to predict the consequence associated with choosing each option at each trial within episode. In other words, participants had to identify the relevant aspects of the stimuli for the goal at hand and rely on their own subjective perception of performance. This derived from their observations of the stimuli presented after each decision and by their own internal assessment criterion which itself was based on their ability to estimate the sum of water (reward value) throughout the trials of each episode.

To summarize, cued performance feedback could have reduced task to simple rule-based learning. Although the optimal strategy consists of a rule-based sequence, the crucial element of the task is that the participant must undergo a phase of exploration in which learning is driven by exploration and assessment of the reward-based consequence associated with each option.

### 3.4 Computational model of consequence

Drift-diffusion models (DDM) have been used to describe how sensory decisions unfold as a function of evidence accumulation (Ratcliff and McKoon, 2008). Likewise, urgency-gating model (Cisek et al., 2009) emphasize the contribution of the passage of time to make sensory based decisions in dynamic environments. Extended versions of the DDM have also been used to describe how evidence relates to value-based decisions via informative saccades (Krajbich et al., 2010; Krajbich and Rangel, 2011), extending into hybrid models that can adapt their parameters over time (Fontanesi et al., 2019; Boelts et al., 2022) via reinforcement learning (Sutton and Barto, 1981). However, these formulations fall short to describe the complexity of brain population dynamics during decision-making and of inhibitory processes therein. Furthermore, they do not capture how action effects and rewards are subjectively perceived and merged in contexts in which these are delayed and must be first perceived and learned, as it occurs in the consequential task. In brief, here we intended a formalization of the neural processes underlying reward-driven, delayed-value, multi-step decisions in a context in which attaining reward is contingent on learning the covert effect of actions on the environment. In this way, learning must operate in the absence of explicit performance feedback, and in the absence of knowledge of the target strategy itself, which is unlike previous RL-based formulations. By contrast, if the purpose of the present study were to merely provide an estimate of the participants’ decisions and learning process, an RL formulation could have been used to solve the credit assignment problem (Minsky, 1961) and learn the behavioral strategy. However, these models fall short of the aforementioned aspects of neuronal dynamics, competition and inhibition that we targeted in this study.

Learning in our model is operationalized by a reinforcement comparison algorithm (Amari, 1998; Brunel and Wang, 2001; Roxin and Ledberg, 2008; Krajbich et al., 2010; Cos et al., 2013; Shahar et al., 2019), scaled by the difference between predicted vs. obtained reward value (Sutton and Barto, 1981; Dayan, 1992), measured accordingly to the participant’s subjectively perceived scale. For simplicity, we assumed a fixed function across participants to quantify reward value [R(T) function in Eq. 4]. Furthermore, to provide the necessary flexibility for the model to capture the full range of participants’ learning dynamics, the model included two free parameters, the learning rate and the decisional uncertainty, to be fit to each participant’s behavior. The result is a model that could faithfully reproduce the full range of behaviors of each participant: RT distribution, pattern of decision-making, and learning time.

The model is organized in three layers. The lower neural dynamics layer represents the average activity of two neural populations competing for selection, each sensitive to one of the two stimuli at each trial. The commitment for an option is made when the difference in firing rate between the two populations crosses a given threshold (Amari, 1998; Brunel and Wang, 2001; Marcos et al., 2013). A similar architecture, with small variations, has been used to model decision-making in a broad set of tasks (Wong and Wang, 2006; Marcos et al., 2013; Marcos and Genovesio, 2016; Lam et al., 2022) and can describe most types of single-trial, binary decision-making, including value-based and perceptual paradigms. Importantly, our model does not provide a clear delineation between deliberation and commitment as DDMs do, but rather a neuron-like unselective ramp-up representation of options that diverge until a commitment is made. Like accumulation-to-bound models, attractor-based models can also account for speed-accuracy trade-offs during decision-making. We chose this kind of formalism because attractor models are more biologically realistic than the abstract accumulation-to-bound ones, and possibly provide a more promising avenue for unifying theories of brain and behavior. This was necessary for our model to provide a plausible explanation for the neural competition and inhibition known to operate in premotor and prefrontal cortical areas. Moreover, our model weighs inputs with recurrent activity during sequences of decisions and projects this formulation for a neighbor neurophysiological study. Note that this layer of the model can be derived analytically from a network of spiking neurons used for making binary decisions (Wang, 2002). Beyond the scope of this study, this model could also subserve probing into working memory (Deco and Rolls, 2005; Wong and Wang, 2006); a transient input could bring the system from the resting state to one of the two stimulus-selective persistent activity states, to be internally maintained across a delay period.

In addition to binary population competition, we claim that modeling consequence-based decision-making requires at least two additional mechanisms. The first one is needed to prioritize a specific policy to guide the decisions; the second one to create an internal mechanism of performance to evaluate these criteria, based on the difference between predicted and obtained reward value. Accordingly, the role of the middle layer (intended decision) is to implement those criteria which in our case depend on the relative value of the stimuli and on the number of trial within episode. Finally, the top layer (strategy learning) carries out learning by reinforcement comparison (Sutton and Barto, 2018) and temporal difference (Sutton and Barto, 1981; Houk et al., 1995).

Altogether, our model introduces a plausible implementation of the neurocognitive processes involved in consequence-based decision-making. Each part of the model is essential to describe decision-making, inhibition, and learning. For the neural dynamics layer, the set of equations corresponds to the most simplified version of a network of brain neurons during binary decision-making (Wong and Wang, 2006); it makes use of only two populations of neurons and a minimal set of parameters. The middle layer consists of one equation (with only one free parameter) and makes use of the simplest possible form of a two-attractor dynamical system (with the addition of a noise component). Finally, the top layer follows a reinforcement comparison algorithm, and adds a single free parameter to the model: the learning rate. Each of these elements is indispensable for a biologically plausible theoretical formalization of consequence-based decision-making. Without the first layer we would not have a biologically plausible decision-making model, without the middle layer we could not describe policy changes, and without the top layer we would not have learning.

Previous research describes models of learning processes during decision-making, for the most part implemented via RL (Sutton and Barto, 2018). Although our paradigm could also be modeled with RL, the clear advantage of our model is that it does not only describe the behavioral patterns of learning for each individual participant, but provides a biophysically plausible description of the underlying brain processes when predicting RTs. Moreover, our model is directly grounded on the neural substrate dynamics, since the mean-field approximation has been derived analytically from networks of spiking neurons (Wang, 2002).

The results and predictions depicted in the model show that the dynamics of the three layers combined can accurately reproduce the behavior of each single participant, including those who did not attain the optimal strategy. The low number (4) of equations in the model, together with the low number of free parameters (7, of which only 3 are used for fitting), makes this model a simple, yet powerful tool able to reproduce a large variety of behaviors. Moreover, unlike the basic RL agents or models for evidence accumulation, our model is biologically plausible and predicting individual behavioral metrics, such as RT, initial bias, and visual discrimination. Note that, for the behavioral part of the model, only one free parameter is used, i.e., learning rate. A larger number of free parameters (at least 3) is needed for classical reinforcement learning algorithms, e.g., Q-learning.

The comprehensive formulation of the model makes it possible to explain and fit various scenarios. We have already mentioned the differences in learning speeds, and that the model could fit any of them, even when there was no learning. Another example is the difference in the order of execution of the blocks. Namely, most participant were able to take the optimal strategy learned in one horizon and generalize it to the other horizon block, making the learning much faster (see Supplementary materials). In our model, this is captured mainly by the initial bias which is calculated for each block individually. As third example, potentially, a characteristic that our model could fit is the difference in RT between trials within episodes and horizons (see Figure 2F). In this manuscript, for simplicity, we decided to perform a single fit for the neural dynamics’ equations, finding one set of parameters per participants. To explain the differences between horizons and trials within episodes, the same fit should be done for each condition. Moreover, even if it is not the case of this specific task, the model is able to adapt in case of a sudden change of strategy. Nevertheless, if this would be the case, it would be advisable to adopt a more realistic adaptation mechanism. Namely, it seems reasonable to assume that, after learning, once a participant realizes that the optimal strategy used so far is not working anymore, he would reset his strategy instead of gradually change it. However, even though it is an interesting topic, this is work for future investigation.

## 4 Conclusion and future work

In this manuscript we have introduced a minimalistic formalism of the brain dynamics of consequence-based decision-making and its associated learning process. We validated this formalism with the behavioral data gathered from 28 human participants, which the model could accurately reproduce. By extending classic, single-trial binary decision-making, we designed a oversight mechanism based on the assessment of the effect of decisions on subsequent stimuli, and a reinforcement rule to modify behavioral preferences. We also designed the consequential task, an experimental framework in which acquiring the most reward value required learning to assess the consequence associated with each option during the decision-making process. Both the experimental results and the model predictions describe consequence-based decision-making as an extended version of value-based decision-making in which the computation of predicted reward value may extend over several trials. The formalism introduces the necessary notions of oversight of the current strategy and of adaptive reinforcement, as the minimal requirements to learn consequence-based decision-making.

Although our model has been designed and tested in the consequential task described here, we argue that its generalization to similar paradigms in which optimal decisions require assessing the consequence associated to the presented options, or sequences of multiple decisions, may be relatively straightforward. Specifically, we envision three possible future extensions to facilitate its generalization. First, the model could incorporate several preference criteria (either simultaneously or combinations thereof) into the intended decision layer: left vs. right or first vs. second, instead of small vs. big, to be determined in a dynamical fashion. This could be achieved with a multi-dimensional attractor model, with as many basins of attraction as the number of preference criteria to be considered.

The second future extension is the re-definition of the reward function R(T) according to the subjective criterion of preference. Namely, a reward value can be perceived differently by different participants, i.e., people operate optimally according to their own subjective perception of the reward value. Because of this, a possible extension is to incorporate an individual reward value function per participant (R(T) in Eq. 4). For simplicity, in this manuscript we set R(T) to be fixed and to be the objective reward value function. In case a participant did not perceive what was the optimal reward value, he/she performed sub-optimally according to objective reward function, and the model responded by allowing the learning constant *k* to be zero. This holds since the optimal strategy was never reached, and the fitting of the participant’s performance was correct. Nevertheless, it remains a standing work of significant interest to investigate different subjective reward mechanisms and their implementation in the model.

Finally, the third enhancement we propose for our model is making the learning rate time dependent, i.e., *k*(*E*). This would facilitate reproducing learning processes starting at different times throughout the session. For example, it is possible that participants initiate the session having in mind a possible (incorrect) strategy and they stick to it without looking for clues, and therefore without learning the optimal policy. Nevertheless, after many trials they may change their mind and begin to explore different strategies. In this case, the learning rate *k*(*E*) would be set to zero for all the initial trials when indeed there is no learning.

Again, we want to emphasize that even if this model is built for the consequential task, it contains all the elements and processes to reproduce behavior from other tasks involving sequential consequence-based decision-making. Note that the strategy learning mechanism is already general enough to adapt to tasks where the optimal policy is not fixed throughout the experiment. In the case of a policy reversal, for example, the learning mechanism would be able to detect a change and adapt accordingly. Finally, we want to stress that our model could be applied to other decision-making paradigms, such as a version of the consequential random-dot task (Britten et al., 1993) or other multiple-option paradigms.

## 5 Materials and methods

### 5.1 Participants

A total of 28 participants (15 males, 13 females; age range 18–30 years; all right hand dominant) participated in the experimental task. All participants were neurologically healthy, had normal or corrected to normal vision, were naive as to the purpose of the study, and gave informed consent before participating. The study was approved by the local Clinical Research Ethics Committee (CEIm Ref. #2021/9743/I) and was conducted in accordance with relevant guidelines and regulations. Participants were paid a €10 show-up fee.

### 5.2 Experimental setup

Participants were situated in the laboratory room at the Facultat de Matemàtiques i Informàtica, Universitat de Barcelona, where the task was performed. The participants were seated in a chair, facing the experimental table, with their chest approximately 10 cm from the table edge and their right arm resting on its surface. The table defined the plane where reaching movements were to be performed by sliding a light computer mouse (Logitech Inc). On the table, approximately 60 cm away from the participant’s sitting position, we placed a vertically-oriented, 24” Acer G245HQ computer screen (1920×1080). This monitor was connected to an Intel i5 (3.20GHz, 64-bit OS, 8 GB RAM) portable computer that ran custom-made scripts, programmed in MATLAB with the help of the MonkeyLogic toolbox, to control task flow (NIMH MonkeyLogic, NIH, United States; https://monkeylogic.nimh.nih.gov). The screen was used to show the stimuli at each trial and the position of the mouse in real time.

As part of the experiment, the participants had to respond by performing overt movements with their arm along the table plane while holding the computer mouse. Their movements were recorded with a Mouse (Logitech, Inc), sampled at 1 kHz, which we used to track hand position. Given that the monitor was placed upright on the table and movements were performed on the table plane (horizontally, approximately from the center of the table to the left or right target side), the plane of movement was perpendicular to that of the screen, where the stimuli and finger trajectories were presented. Data analyses were performed with custom-built MATLAB scripts (The Mathworks, Natick, MA), licensed to the Universitat de Barcelona.

### 5.3 Consequential decision-making task

This section describes the consequential decision-making task, designed to assess the role of consequence on decision-making while promoting prefrontal inhibitory control (Wessel and Aron, 2017). Since consequence depends on a predictive evaluation of future contexts, we designed a task in which trials were grouped together into episodes (groups of one, two or three consecutive trials), establishing the horizon of consequence for the decision-making problem within that block of trials.

The number of trials per episode equals the horizon *n_H_* plus 1. In brief, within an episode, a decision in the initial trial influences the stimuli to be shown in the next trial(s) in a specific fashion, unbeknown to our participants. Although a reward value is gained by selecting one of the stimuli presented in each trial, the goal is not to gain the largest amount as possible per trial, but rather per episode.

Each participant performed 100 episodes for each horizon *n_H_* = 0, 1, and 2. In the interest of comparing results, we have generated a list of stimuli for each *n_H_* and used it for all participants. To avoid fatigue and keep the participants focused, we divided the experiment into 6 blocks, to be performed on the same day, each consisting of approximately 100 trials. More specifically, there was 1 block of *n_H_ = 0* with 100 trials, 2 blocks of *n_H_ = 1* each with 100 trials, and 3 blocks of *n_H_ = 2* with two of them of 105 trials and one of 90. Finally, we have randomized the order in which participants performed the horizons.

Figure 1 shows the timeline of one *n_H_* = 1 episode (2 consecutive trials). The episode consists of two dependent trials. At the beginning of the trial, the participant was required to move the cursor onto a central target. After a fixation time (500 ms), the two target boxes were shown one after the other (for 500 ms each) to the left and right of the screen, in a random order. Targets were rectangles filled in blue by a percentage corresponding to the reward value associated with each stimulus (analogous to water containers). Next, both targets were presented together. This served as the GO signal for the participant to choose one of them (within an interval of 4 s). Participants had to report their choice by making a reaching movement with the computer mouse from the central target to the target of their choice (right or left container). If the participant did not make a choice within 4 s, the trial was marked as an error trial. Once one of the targets had been reached for and the participant had held that position (500 ms), the selection was recorded, and a yellow dot appeared above the selected target, indicating successful selection and reward value acquisition. In case of horizons larger than 0, the second trial started following the same pattern, although with a set of stimuli that depended on the previous decision (see next section). A progress bar at the bottom of the screen indicates the current trial within the episode (for *n_H_* = 1, 50% during the first trial, 100% during the second trial).

At the beginning of the session, participants were given instructions on how to perform the task. Specifically, using some sample trials, we demonstrated them how to select a stimulus by moving the mouse. Step by step we showed that a target appears in the center of the screen indicating the start of an episode. We told them that they had 4 s to move the cursor to the central cross. After moving the cursor to the central cross, two bars appear, one after the other, and once both appear together/simultaneously, they had 4 s to make their decision by moving the cursor over one of the two bars. At that point a yellow dot appears over the bar indicating their selection. After that, the central target appears again indicating the beginning of a new trial. After explaining how to technically execute the task, we focused on explaining the task goal. We showed them a schematic of the task, much like the one in Figure 1A illustrating the structure of trials and episodes. We told them that the goal is to get as much reward (water) as possible in each episode, and that for episodes with more than 1 trial each, the choice in a trial may have an effect on what appears in the next trial in the same episode. We encouraged them to explore in order to try to figure out what that effect might be, while keeping in mind that their goal is always to maximize the total reward in each episode. Finally, we told them that they will be presented with a series of episodes in a row, each episode is independent, meaning that their decisions in one episode have no effect on subsequent ones.

### 5.4 Episode structure

The participants were instructed to maximize the cumulative reward value throughout each episode, namely the sum of water contained by the selected targets across the trials of the episode. If trials within an episode were independent, the optimal choice would be to always choose the largest stimulus. Since one of the major goals of our study was to investigate delayed consequence assessment involving adaptive choices, we deliberately created dependent trial contexts in which making incentive decisions (selecting the larger stimulus) would not lead to the most cumulative reward value within episode.

To promote inhibitory choices, the inter-trial relationship was designed such that selecting the small (large) stimulus in a trial, yielded an increase (decrease) in the mean value of the options presented in the next trial. As explained below, because of the parameters choice we made, always choosing the larger stimulus did not maximize cumulative reward value for *n_H_ = 1, 2.*

Trials were generated according to 3 parameters: horizon’s depth *n_H_*, perceptual discrimination (in terms of difference ΔS between the stimuli), and the gain/loss *G* in mean size of stimuli for successive trials. The stimuli $s_{1,2}$ presented on the screen could take values ranging from 0 to 1. Trials were divided into five difficulty levels by setting the difference between stimuli ΔS∈{0.01,0.05,0.1,0.15,0.2} .

For horizon *n_H_ = 0*, for each trial the stimuli $s_{1,2}$ are generated as to have mean *M* and difference *d* between them, i.e., $s_{1,2}=M\pm \Delta S/2$ . To have stimuli ranging from 0 to 1, the mean *M* is randomly generated using a uniform distribution with bounds $\left[\Delta S_{\max}/2,1-\Delta S_{\max}/2\right]$ , where $\Delta S_{\max}=0.2$ is the maximum ΔS. In horizon *n_H_ = 1*, each episode consists of 2 dependent trials. Specifically, the stimuli presented in the second trial depend on the selection reported in the previous trial of that same episode. More specifically, the rule is such that if the choice of the first trial is the smaller/larger stimulus, the mean of the pair of stimuli in the second trial will be increased/decreased by a specific gain *G*. In practice, the first trial of an *n_H_ = 1* episode is generated in the same way as for horizon *n_H_ = 0*, i.e., the two stimuli equal $s_{1,2}=M\pm \Delta S/2$ . The stimuli in the second trial within the same episode could be either $s_{1,2}=M+G\pm \Delta S/2$ or $s_{1,2}=M-G\pm \Delta S/2$ , depending on the previous decision. Note that the difficulty of the trial remains constant within episode. A schematic for the trial structure is shown in Figure 1. Again, to have stimuli ranging from 0 to 1, the mean *M* is randomly generated using a uniform distribution with bounds $\left[G+\Delta S_{\max}/2,1-G-\Delta S_{\max}/2\right]$ . In horizon *n_H_ = 2*, episodes consist of three trials. The trial generation is structured as for horizon *n_H_ = 1*. Namely, the first trial has stimuli $s_{1,2}=M\pm \Delta S/2$ , the second $s_{1,2}=M\pm G\pm \Delta S/2$ , and the third $s_{1,2}=M\pm G\pm G\pm \Delta S/2$ . To have stimuli ranging from 0 to 1, the mean *M* is randomly generated from a uniform distribution with bounds $\left[2G+\Delta S_{\max}/2,1-2G-\Delta S_{\max}/2\right]$ . We set the gain/loss parameter to *G = 0.3* and *G = 0.19* for horizon *n_H_ = 1* and *n_H_* = 2, respectively. Our choice was motivated by the fact that G should be big enough to have a deterministic optimal strategy, i.e., always choosing the smaller reward value apart from the last trial within episode. In other words, choosing the bigger stimulus never compensates for the loss given by G. Moreover, *G* should be big enough to let the participants perceive the gain/loss between trials, while simultaneously allowing some variability for the randomly generated means *M*.

### 5.5 Statistical analysis

The dependency of PF and RT on VD together with the other variables must be established statistically. To assess the learning process, we quantified the relationship of PF and RT with horizon *n_H_*, trial within episode *T_E_*, and episode *E*. To obtain consistent results, we adjusted these variables as follows. In the calculation, the trial within episode is reversed, from last to first, because the optimal choice for the last *T_E_* (large) is the same regardless of the horizon number. Furthermore, regarding the model for PF, to consider trials within episode independently, we adapted the notion of PF (defined as a summary measure per episode) to an equivalent of PF per trial, i.e., the probability of choosing the optimal choice $P_{oc}$ . Finally, to assess the difference between learning groups, we introduce the categorical variable L that identifies the group of participants that learned the optimal strategy and the ones who did not, according to Figure 2A. We then used a generalized linear mixed effects model (Verbeke and Molenberghs, 2009; Gałecki and Burzykowski, 2013) to predict PF and RT. The independent variables for the fixed effects are horizon *n_H_*, trial within episode $T_{E}$ , and the passage of time expressed in terms of episodes *E,* and ΔS. We set the random effects for the intercept and the episodes grouped by participant *p*; we write the random effects as (E|p) . The resulting models are: $P_{oc}\sim L\cdot E+L\cdot \Delta S+L\cdot n_{H}\cdot T_{E}+(E|p)$ and $RT\sim L\cdot E+L\cdot \Delta S+L\cdot n_{H}\cdot T_{E}+(E|p)$ . The results of the statistical analysis are reported in Table 2. The regression coefficients, with their respective group significance, are shown in Figures 2E,F.

**Table 2:** Linear mixed effects model for the percentage of optimal choices selected $P_{oc}$ and for the reaction time RT .

	$P_{oc}\sim L\cdot E+L\cdot \Delta S+L\cdot n_{H}\cdot T_{E}+(E|p)$	$RT\sim L\cdot E+L\cdot \Delta S+L\cdot n_{H}\cdot T_{E}+(E|p)$
F-stat.	175	205.9
*p*-value	0	0

**Fixed effects**	**Estimate**	**SE**	***t* Stat**	***p* Val**	**Lower**	**Upper**	**Estimate**	**SE**	***t* Stat**	***p* Val**	**Lower**	**Upper**
Intercept	6.35	0.40	15.6	10^−54^	5.55	7.15	0.75	0.15	4.95	10^−07^	0.456	1.05
$T_{E}$	4.38	0.26	−16.9	10^−64^	−3.88	4.89	−0.58	0.08	−7.04	10^−12^	−0.75	−0.42
$n_{H}$	−1.55	0.18	−8.35	10^−17^	−1.92	−1.18	−0.48	0.06	−8.36	10^−17^	−0.60	−0.37
*E*	−0.001	0.003	−0.40	0.69	−0.01	0.01	−0.05	0.04	−1.21	0.23	−0.13	0.03
ΔS	−1.10	0.67	−1.65	0.10	−2.40	0.21	−0.24	0.02	−15.78	10^−55^	−0.27	−0.21
*L_1_*	−2.31	0.47	−4.90	10^−7^	−3.23	−1.39	−1.45	0.17	−8.42	10^−17^	−1.79	−1.11
$T_{E}:n_{H}$	−1.21	0.14	8.52	10^−17^	−1.49	−0.93	0.36	0.04	8.21	10^−16^	0.28	0.45
$T_{E}:L_{1}$	−2.05	0.29	6.97	10^−12^	−2.63	−1.47	1.11	0.09	11.89	10^−32^	0.93	1.30
$n_{H}:L_{1}$	−0.08	0.21	−0.37	0.71	−0.51	0.35	0.62	0.07	9.48	10^−21^	0.49	0.75
$E:L_{1}$	0.02	0.003	4.88	10^−6^	0.01	0.02	−0.02	0.04	−0.49	0.62	−0.11	0.07
$\Delta S:L_{1}$	8.61	0.78	11.08	10^−28^	7.08	10.13	−0.06	0.02	−3.42	10^−3^	−0.09	−0.02
$T_{E}:n_{H}:L_{1}$	−0.22	0.16	−1.40	0.16	−0.54	0.09	−0.51	0.05	−10.31	10^−25^	−0.61	−0.42

The independent variables for the fixed effects are horizon n_H_, trial within episode $T_{E}$ , and the passage of time expressed as episodes E, and ΔS. We set the random effects for the intercept and the episodes grouped by participant *p*.

## Data availability statement

The datasets generated during and analyzed during the current study are available in the eBrains repository, https://search.kg.ebrains.eu/instances/0d145ebe-3ecd-4b3c-9400-913a8cd21a6a. The codes generated during the current study are available in the eBrains repository https://search.kg.ebrains.eu/instances/ffda985e-9023-4d06-aa79-0ec7109ff55c linked to the GitHub repository https://github.com/gloriacec/Model_ConsequenceBasedDecisionMaking.

## Ethics statement

The studies involving humans were approved by Clinical Research Ethics Committee (CEIm Ref. #2021/9743/I). The studies were conducted in accordance with the local legislation and institutional requirements. The ethics committee/institutional review board waived the requirement of written informed consent for participation from the participants or the participants’ legal guardians/next of kin because all participants were neurologically healthy, had normal or corrected to normal vision, were naive as to the purpose of the study, and gave informed consent before participating.

## Author contributions

GC: Conceptualization, Data curation, Formal analysis, Investigation, Methodology, Software, Validation, Visualization, Writing – original draft, Writing – review & editing. MD: Data curation, Investigation, Methodology, Writing – review & editing. EB: Investigation, Writing – review & editing. MA: Investigation, Writing – review & editing. SR: Writing – review & editing. PP: Writing – review & editing. SF: Funding acquisition, Supervision, Writing – review & editing. AD: Funding acquisition, Supervision, Writing – review & editing. RM-B: Funding acquisition, Supervision, Writing – review & editing. IC: Funding acquisition, Investigation, Methodology, Supervision, Writing – original draft, Writing – review & editing.
