# Supplementary material for: Cognitive mechanisms of learning in sequential decision-making under uncertainty: an experimental and theoretical approach
Source: Front Behav Neurosci. 2024 Aug 12;18:1399394. doi: 10.3389/fnbeh.2024.1399394 (PMC11346247; doi:10.3389/fnbeh.2024.1399394)
Supplement: Supplementary file 1 [file Data_Sheet_1.pdf]

SUPPLEMENTARY MATERIALS

Cognitive mechanisms of learning in sequential decision-making under uncertainty: An experimental and theoretical approach

Gloria Cecchini[1,2], Michael DePass[2], Emre Baspinar[3], Marta Andujar[4], Surabhi Ramawat[4], Pierpaolo Pani[4], Stefano Ferraina[4], Alain Destexhe[3], Rubén Moreno-Bote[2,5], Ignasi Cos[1,5]

[1] *Facultat de Matemàtiques i Informàtica, Universitat de Barcelona, Barcelona, Catalonia, Spain*
[2] *Center for Brain and Cognition, DTIC, Universitat Pompeu Fabra, Barcelona, Catalonia, Spain*
[3] *CNRS, Paris-Saclay University, Institute of Neuroscience (NeuroPSI), Saclay, France*
[4] *Department of Physiology and Pharmacology, Sapienza University of Rome, Rome, Italy*
[5] *Serra-Hunter Fellow Programme, Barcelona, Catalonia, Spain*

Corresponding Author: gloria.cecchini@ub.edu

Exploratory strategy

We analyzed the exploratory strategy participants used. In particular, we tested whether participants only considered the size of the stimuli (small/big), or if they also tried other hypotheses, such as the order of presentation (first/second) or the location (left/right) of the stimuli. The result of this analysis is shown in FigSupp 1, which depicts the proportion of trials for three exploratory strategies, i.e., size, location, and order of presentation.

Participants mostly considered the size as a possible factor for optimization during the trials when the optimal strategy was not yet found, FigSupp 1a. These panels show that some participants explored the location and the order of appearance. Nevertheless, according to a questionnaire we ran at the end of each session, asking participants what strategy they learned and/or tried, only 4 mentioned the order of appearance, and none the location.

From the 6 participants who did not learn the optimal strategy (FigSupp 1b), only 1 considered the order of appearance throughout the whole session. This participant reported in the questionnaire that they used a complex combination of appearance and size as metric for optimization. Another participant reported in the questionnaire that they performed at random for $n_H=0$, and for $n_H=1,2$ tried to make the cumulative reward fill to the top of the bar, resulting in choosing small-big for $n_H=1$, and small-small-big for $n_H=2$. All other 4 participants repeatedly chose the larger stimulus for all trials.

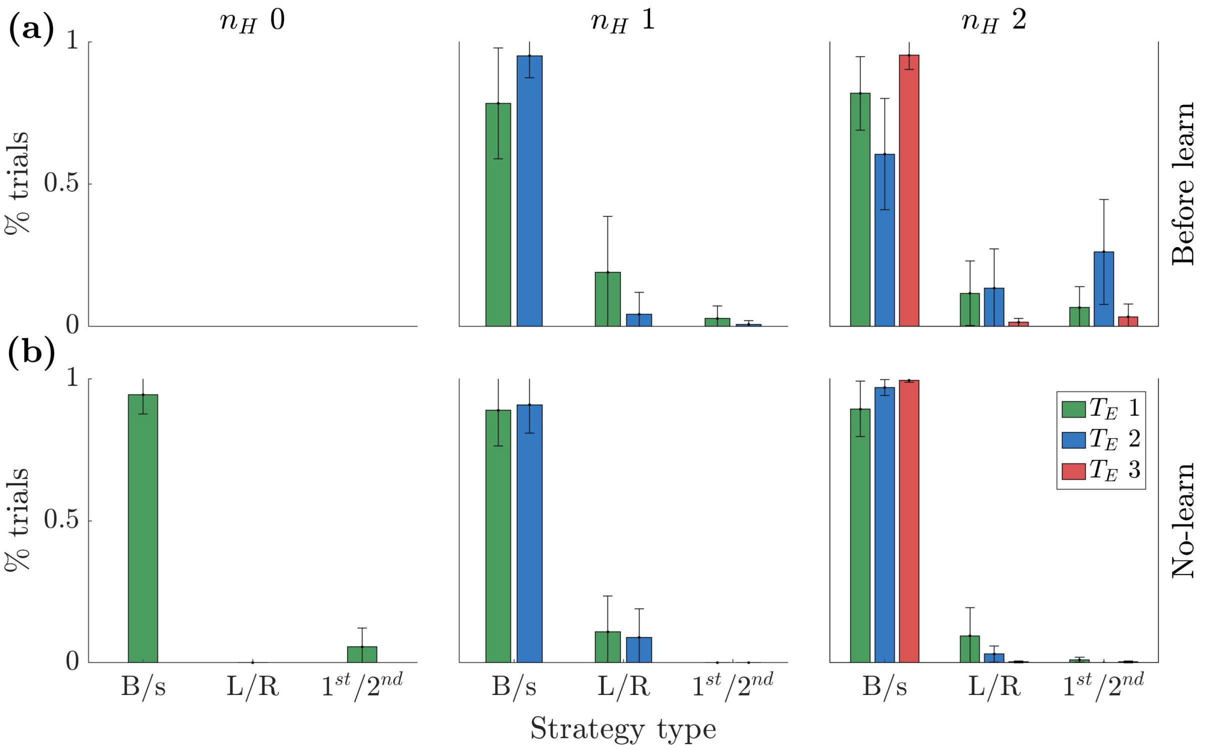

*FigSupp 1. Exploratory strategy during the episodes before learning and for the participants who did not learn the optimal strategy. **(a)** Participants mostly considered the size as a possible factor for optimization during the trials when the optimal strategy was not yet found. For n_H 0 there are not enough trials before learning to perform this analysis. **(b)** From the 6 participants who did not learn the optimal strategy only one considered the order of appearance throughout the whole session.*

Learning time and order of performance of different horizons

In Materials and Methods, Sec. Consequential Decision-Making task, we described the structure of the task, and in particular we mentioned that we randomized the order in which participants performed the horizons. This means that, for example, some participants performed n_H 2 before n_H 0. We wondered if the order of the horizons had an influence on the learning time. For example, were the participants who started with n_H 1 faster in learning the optimal strategy than the ones who started with n_H 2? To address this, we performed a thorough investigation analyzing the learning time for each block of recording of the session.

FigSupp 2 shows the learning time for each block in order of execution (x-axis). Each plot is a different participant. On the y-axis, the learning time is reported in terms of episodes, and above 50 means no-learning. Different horizons are depicted with different markers and colors: n_H 0 green circle, n_H 1 blue triangle, n_H 2 red cross. This figure also shows that 2 participants did not learn any horizon, 4 participants learned n_H 0, but not either n_H 1 or n_H 2, and the remaining 22 managed to learn at least one of n_H *1, 2*. Note that participants 1, 2, and 3 in the main text of the manuscript correspond to participants 20, 6, and 7 in FigSupp 2, respectively.

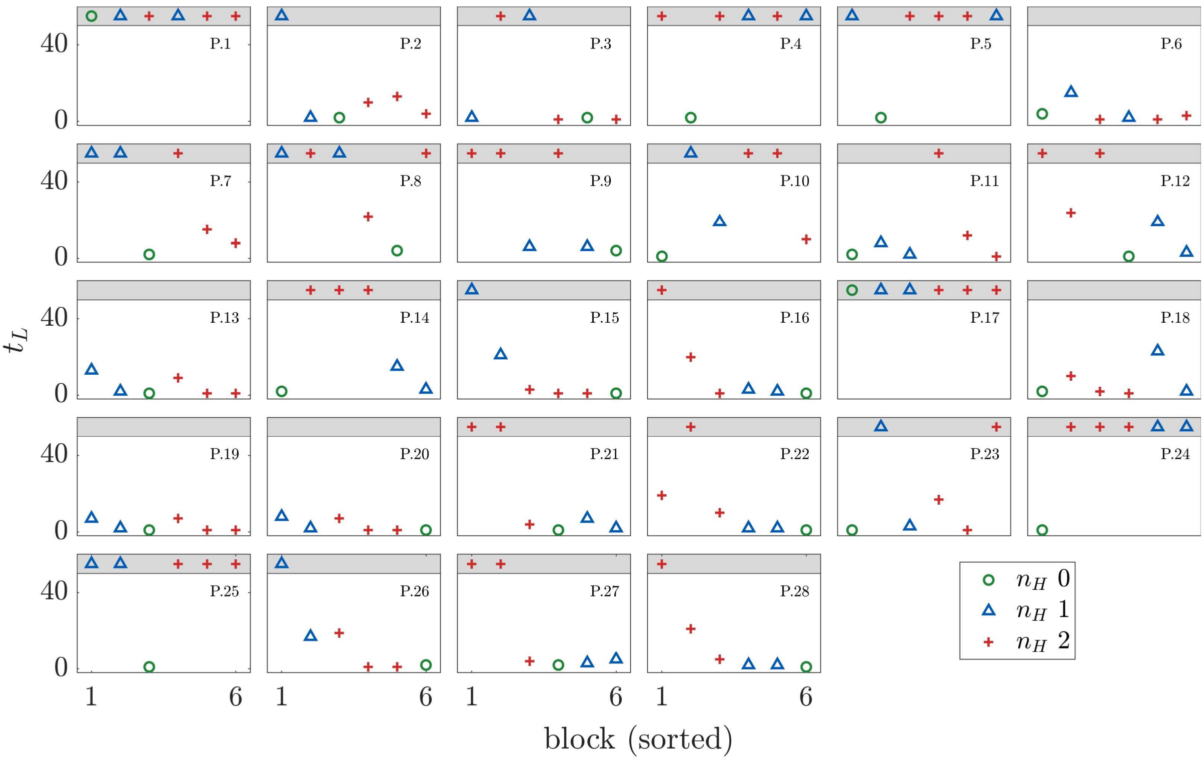

FigSupp 2. Learning time per block of recording in order of execution. Each plot is a different participant. On the y-axis, the learning time is reported in terms of episodes (above 50 means no-learning). Different horizons are depicted with different markers and colors (n_H 0 green circle, n_H 1 blue triangle, n_H 2 red cross). Two participants did not learn any horizon, 4 participants learned n_H 0, but not either n_H 1 or n_H 2, and the remaining 22 learned at least one of n_H 1, 2.

Interestingly, FigSupp 2 shows that most participants, when they had learned the optimal strategy in one horizon, they then generalized and learned the optimal strategy in the next horizon much quicker. This collective result is presented in FigSupp 3, here we excluded the 2 participants who did not learn n_H 0. The learning time is shown on the y-axis, each green dot refers to a participant, and the three plots refer to different horizons. In other words, the following plots show the learning time for each of the three horizons separately. Moreover, they show the difference if that specific horizon was performed before or after other conditions. Namely, panel (a) depicts the learning time in n_H 0 in the case it was the first block carried out, or not. If n_H 0 was performed during the first block, participants took a little longer to learn the optimal strategy than if they had already performed n_H 1 or 2 before. Panel (b) illustrates the learning time in n_H 1, separating the participants according to if n_H 1 was performed before or after n_H 2. Finally, panel (c) portrays the learning time in n_H 2, separating the participants according to if n_H 2 was performed before or after n_H 1. Note that these plots show all participants, including the ones that never learned n_H 1, 2, but did learn n_H 0. From the results in (b-c), we can conclude that, in the horizons with consequence (i.e., n_H 1, 2), participants needed less time to learn the optimal strategy of one horizon, if they had already performed the other one. We speculate that once the optimal strategy in any consequential block was understood, participants generalized the rule and by abstraction applied it to the other horizon.

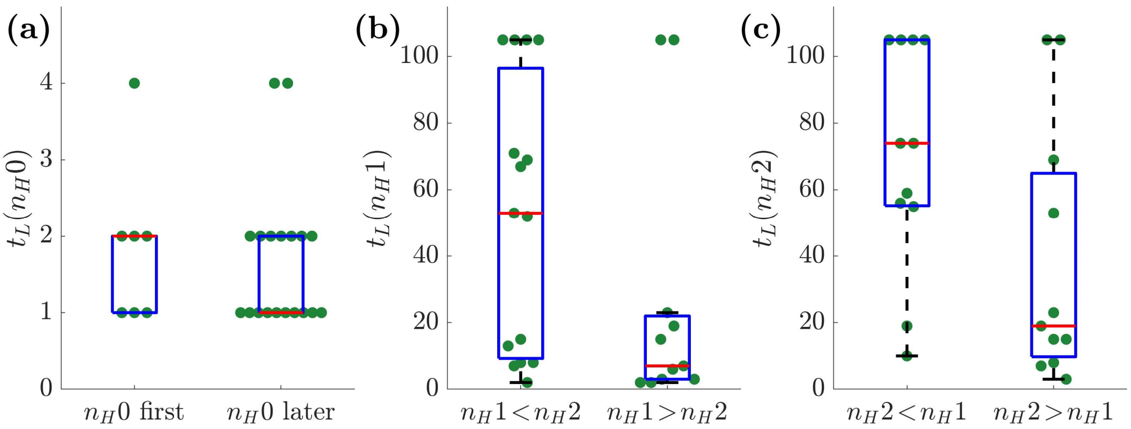

FigSupp 3. Learning time as a function of the horizon, and the order of execution. The 2 participants who did not learn n_H 0 were excluded from this analysis. Green dots refer to participants, and the three panels refer to different horizons. ***(a)*** *learning time in* n_H *0 in the case it was the first block carried out (left), or not (right).* ***(b)*** *Learning time in* n_H *1, for the participants that performed* n_H *1 before (left) or after (right)* n_H *2.* ***(c)*** *Learning time in* n_H *2, for the participants that performed* n_H *2 before (left) or after (right)* n_H *1.*

Parameter space analysis

To help reading Figure 7, FigSupp 4 shows the dependency of RT with the free parameters of Eq. 3. When not varied for the plot, we fixed the parameters to τ=67, Δ=22 Hz, and σ=0.001 in panels (a-c), and τ=39, Δ=26 Hz, and σ=0.005 in panels (d-f). Both the mean and standard deviation increase consistently with both the time constant τ and the threshold Δ . The noise intensity σ does not have a substantial influence on RT.

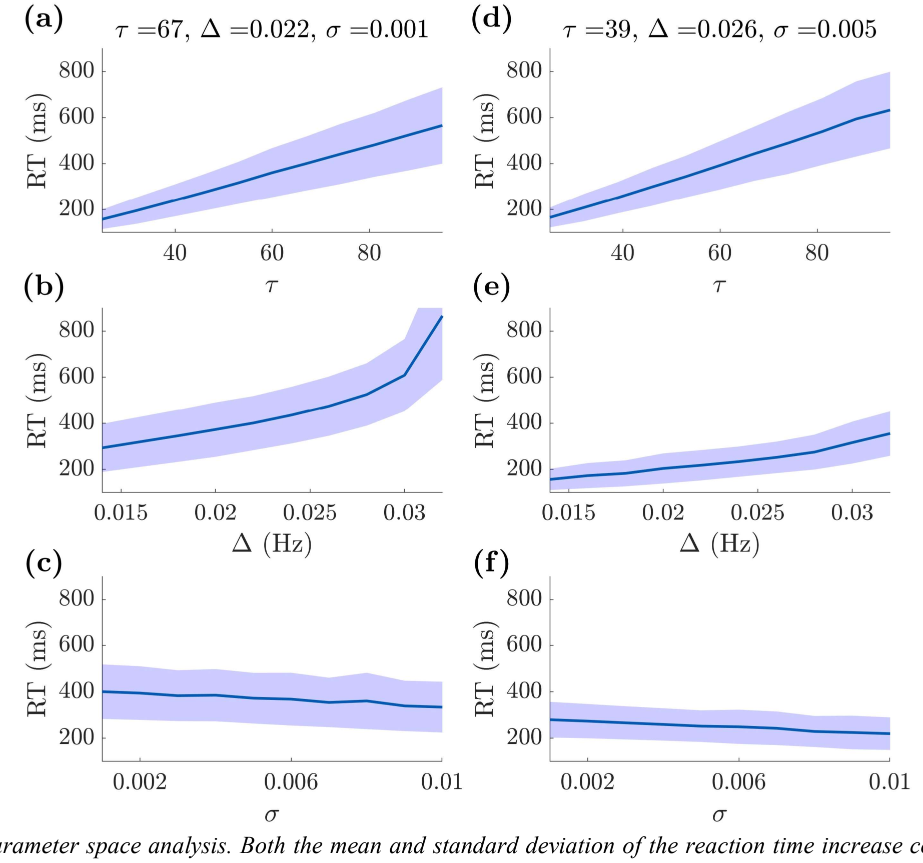

FigSupp 4. Parameter space analysis. Both the mean and standard deviation of the reaction time increase consistently with both ***(a,d)*** *the time constant τ and* ***(b,e)*** *the threshold Δ.* ***(c,f)*** *The noise intensity σ does not have a substantial influence on the reaction time. – Parameters used:* ***(a-c)*** *τ=67, Δ=22 Hz, and σ=0.001;* ***(d-f)*** *τ=39, Δ=26 Hz, and σ=0.005, when not varied for the plot.*

Parameters recovery analysis

To test the robustness of the fitting method, and to test it against overfitting, we performed a parameter recovery analysis (73–75). FigSupp 5 a-c show the relationship between the true parameters and the recovered ones for τ, β, and *k*. The corresponding correlation coefficients are *r=0.994* (*p-val=0*), *r=0.9941* (*p-val=0*), and *r=0.9104* (*p-val=0*) for τ, β, and *k* respectively. We obtained correlation coefficients close to 1, which reflect an excellent recovery.

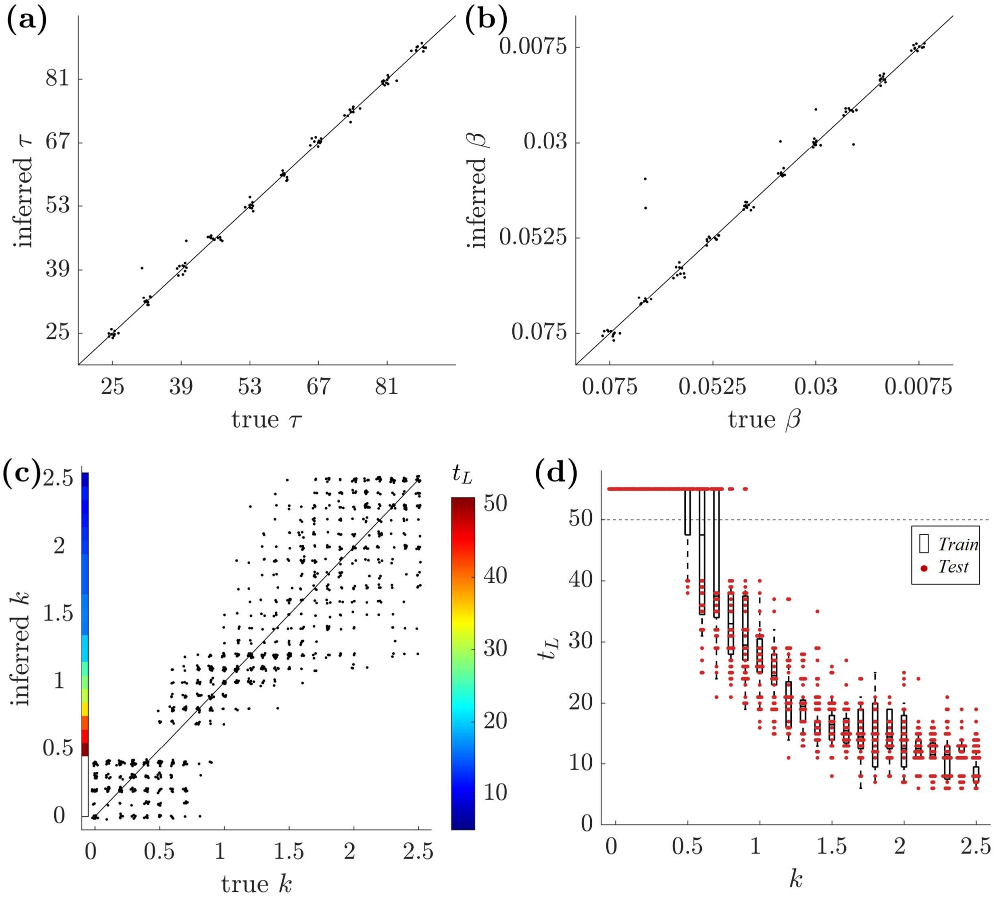

FigSupp 5. Parameters recovery analysis. ***(a-c)*** *Relationship between the true parameters and the recovered ones for τ, β, and* k. ***(d)*** *Relationship between the parameter for the learning rate k and the learning time, for the train and test sets.*
